# The impact of coastal erosion on the archaeology of the Cyrenaican coast of Eastern Libya

**DOI:** 10.1371/journal.pone.0283703

**Published:** 2023-04-12

**Authors:** Kieran Westley, Julia Nikolaus, Ahmad Emrage, Nic Flemming, Andrew Cooper

**Affiliations:** 1 School of Geography & Environmental Sciences, Ulster University, Coleraine, United Kingdom; 2 Department of Archaeology, University of Benghazi, Benghazi, Libya; 3 Independent Researcher, United Kingdoms; Austrian Academy of Sciences, AUSTRIA

## Abstract

Coastal erosion in Cyrenaica (Eastern Libya) represents a major problem for archaeology and heritage management. The area is rich in archaeological sites, often understudied or not fully documented, but also has extensive stretches of vulnerable eroding coastline. This study demonstrates the extent and impact of erosion via shoreline change assessment at two spatial scales. Firstly, wide area assessment using shorelines extracted from a time-series of medium-resolution Landsat imagery. Secondly, site-specific assessment using recent and historic Very High Resolution (VHR) satellite imagery. In both cases, extracted shorelines at different timesteps were compared using the Digital Shoreline Analysis System (DSAS) tool to quantify rates and magnitudes of shoreline movement. The results show extensive zones of erosion at and around the key ancient harbour sites of Apollonia, Ptolemais and Tocra. They also suggest increased rates of coastal retreat in recent years, which is likely linked to anthropogenic actions such as sand mining and urbanization. Forecasts based on present-day shoreline change rates, coupled with ground-level documentation of the vulnerable shorelines is used to identify archaeological features and structures which will likely be progressively damaged or destroyed over the next 20 years. The ability to actively protect archaeological sites is unclear, but there is a clear need for mitigation in the form of enhanced awareness of environmental problems (e.g. caused by sand mining) and more intensive survey/documentation of sites and areas which will be lost in the coming years.

## Introduction

The coastline of Cyrenaica (Eastern Libya), running between the Gulf of Sirte and the present day Egypt-Libya border, has a long history of human occupation stretching back into the Palaeolithic [1,2]. Minoan pottery found in Cyrenaica suggests regular contact across the eastern Mediterranean already in the early Bronze Age. Greek settlers arrived in the seventh century BC and established the large inland settlement of Cyrene and the coastal towns of Apollonia (the harbour of Cyrene), Ptolemais, Taucheira (Tocra) and Euesperides (modern Benghazi). The region was under Ptolemaic rule from 322 BC to 96 BC, after which it fell under Roman control, becoming a province in 31 BC and then part of the Byzantine Empire after AD 395. The Arab conquest of Cyrenaica (AD 642–645) eventually led to a breakdown of the maritime trade links which had operated through the Roman and Byzantine periods, and the successful coastal port cities fell into decline [3,4].

This rich and varied history of occupation has left behind numerous archaeological sites. Unfortunately, many are at risk from a range of threats. These include unregulated building activities, clearance, sand mining and coastal erosion [5]. The latter has been documented on the Cyrenaican coast since the early 19^th^ century [6] and recent reports highlight its destructive impact on archaeological sites at the water’s edge, notably at Tocra and Apollonia [4,7–9]. In addition to the best-studied large sites of Apollonia, Tocra and Ptolemais, there are many smaller sites along the Cyrenaican shore. Many of these are presently unstudied but they are also at risk from erosion [5,10,11]. This is all the more worrying considering that coastal erosion and flooding are projected to increase through the 21^st^ century because of climate change and sea-level rise (SLR) [12–16]. This could be further exacerbated by human action, such as reduced sediment supply caused by upstream damming, canalization of rivers/wadis and urbanization [17,18]. Moreover, modelled wave regimes also suggest that Cyrenaica is more prone to persistent wave attack in comparison with other regions of the Mediterranean coast [19]. Consequently, this shoreline can be regarded as highly vulnerable from an archaeological perspective.

Understanding the location and severity of these impacts requires accurate baseline data on past and present coastal change. Ideally, this would be done at sub-regional to site-scales, given that coastal change can be complex and varies with local wave climate, topography, geomorphology and sediment supply. Progress has been made in detailed data collection and vulnerability assessment in some countries [20,21], but is lacking for Eastern Libya. Existing broad-scale archaeological vulnerability assessments have either not considered Cyrenaica owing to a focus on World Heritage Sites [22,23] or have relied heavily on outputs from global-scale models [24]. Although these types of study are important in raising awareness of the issues at stake and providing generalized insights into spatio-temporal patterns of vulnerability, it is an open question as to how well they downscale to the sub-national and site scales at which practical heritage management actually takes place [25]. On the other hand, although eyewitness observations of erosion exist for this area (e.g. [8]), these have historically been intermittent, albeit increasing, thanks to the work of the Cyrenaica Coastal Survey (CCS) [5]. Consequently, there is a need to set these observed snapshots of impact within the overall context of longer-term and regional-scale patterns of change.

In this paper we attempt to bridge the gap between global-to-regional scale studies and intermittent ground-level observations. This is done by leveraging the growing global archive of high- to medium-resolution satellite imagery in order to quantify trends in 20^th^ century shoreline change at the key Cyrenaican coastal sites of Tocra, Apollonia and Ptolemais. We set these in the context of local- to regional-scale coastal geomorphology and past relative sea level (RSL) change, suggest drivers for observed impacts and tentatively identify future trends. The ultimate intention is to provide assessments at a scale which are appropriate to feed into future management and research plans for these endangered archaeological sites.

## Study area

### Physical geography and geology

**Fig 1:**
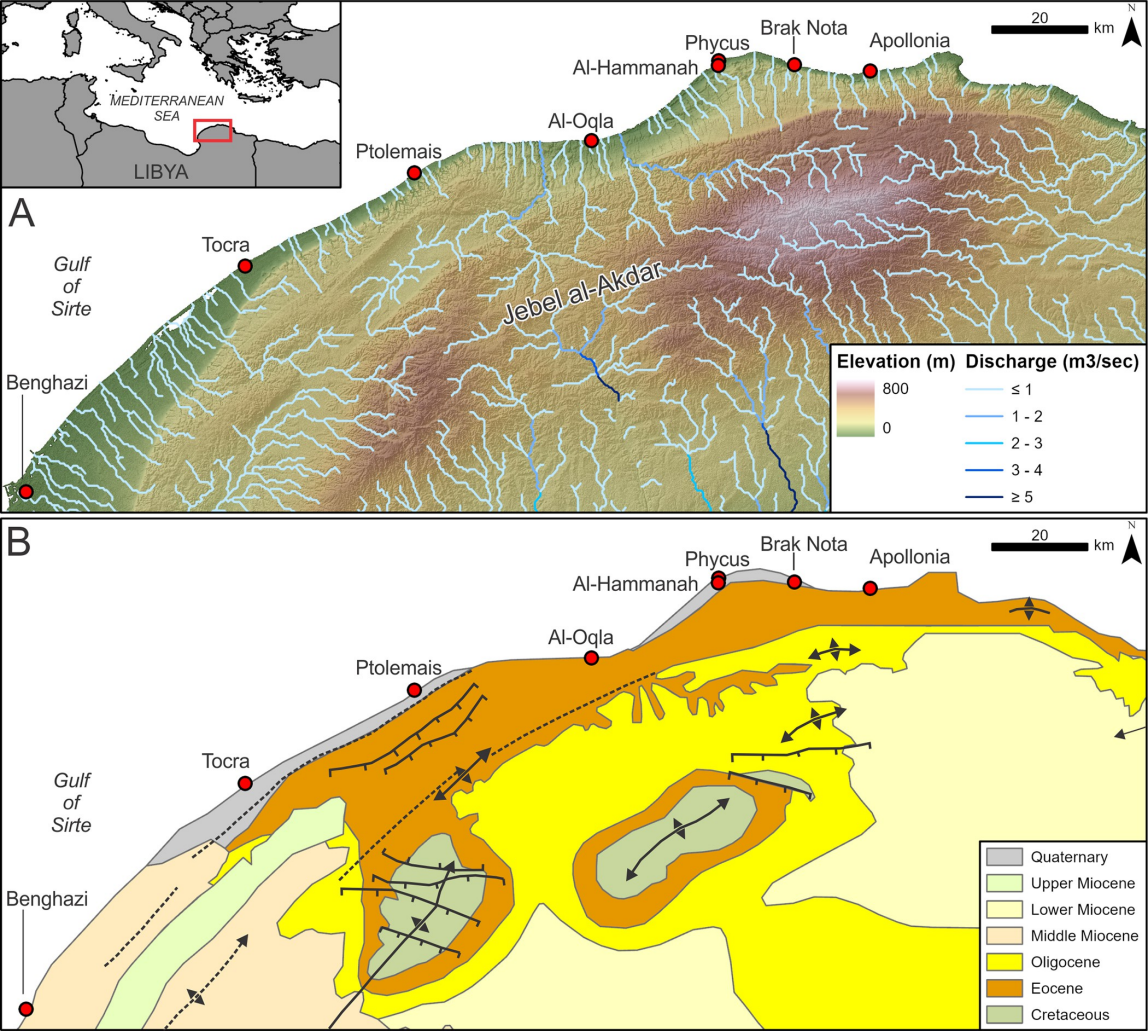
Study area topography and key placenames. **A)** Elevation above sea-level from the NASA Digital Elevation Model (DEM [29]) and overlaid with local fluvial systems as represented by the HydroSHEDS Free Flowing Rivers dataset [30] B) Geological map of the study area (modified from [27]).

The study area lies within the North Cyrenaica Inverted Basin, an uplifted succession of sedimentary rocks originally deposited in a basin setting from the Middle Jurassic to the Lower Cretaceous. Uplift began in the Upper Cretaceous, driven by the converging African, European and Aegean tectonic plates and ultimately resulted in the creation of the Jebel al-Akdar anticline. This mountain range reaches up to ~880 m elevation and forms the southern border of the Cyrenaica coastal plain. Exposed bedrock on the north-facing slopes of the Jebel al Akdar consists mainly of Eocene limestone between Tocra and Derna with Miocene limestones outcropping south of Tocra and around Benghazi [26,27]. Two escarpments run around the coast-facing slopes of the Jebel al-Akdar [28]. The lower escarpment is at ~200 m elevation and, to the west, lies ~20–50 km inland of the present shoreline creating a gradual slope to the coast. Moving east, slopes steepen and the coastal plain gradually narrows, such that the lower escarpment is roughly 2 km inland at Ptolemais. The narrow coastal strip backed by steep slopes, cliffs and escarpments continues until the Gulf of Bomba (Fig 1).

Mean tidal range is generally <0.15 m [31] and coastal processes are dominated by wind and waves. The dominant wind direction is overwhelmingly from the north and northwest. For example, at Benghazi, winds from the northwest to north-northeast quadrant blow for approximately 64% of the year [32]. These dominant winds create waves principally from these directions, striking directly onto the coast perpendicularly for part of its length. *In situ* wave records are not available. However, hindcast modelling for Mediterranean Basin scale indicates the following [19]. For average winter conditions (November–March) the significant wave height (*Hs*) reaching the coastline is 1.25 m for the 50^th^ percentile, and >4 m for the 99^th^ percentile. These can be respectively regarded as representative of average and extreme conditions. Summer (June–August) *Hs* is lower: 0.75 m for the 50^th^ percentile and 2 m for the 99^th^ percentile ([19]). Similarly, maximum individual wave heights (*Hmax*) during winter range from 2.5–8 m and during summer from 1.75–5 m. In both cases, the range reflects the 50^th^ and 99^th^ percentiles ([19]).

Coastal geomorphology comprises mainly sand beaches backed by low slopes or unconsolidated cliffs between Benghazi and Ptolemais. The curve and orientation of the coast in this sector is such that the dominant northerly wind tends to shift sediments southwards. The section of coastline from around Ptolemais to Apollonia is approximately normal to the wind direction. Longshore transport, if it exists, tends, on average, to denude the coast of sediments towards the southern and eastern flanks. The irregular nature of the shore, with low cliffs, headlands and platforms tends to trap small quantities of sediment in the form of pocket beaches. There are no major rivers, only a series of often deeply-incised wadis which flow only seasonally or intermittently.

### Sea-level change

The study area contains evidence of both high and low sea level. Quaternary uplift is evidenced by a series of up to six erosional cliffs and terraces locating on the seaward slopes of the Jebel al-Akdar. These can be traced individually at consistent elevations up to 140–190 m above present sea level over distances of several kilometres. In places, the terraces and cliff bases are covered by calcarenite deposits of marine or aeolian origin [27,28,33–35]. These are interpreted as Pleistocene shorelines given their similarity to other uplifted features around the Mediterranean (e.g. [36,37]). Precise dating is not available beyond the possible assignation of the lowest terrace to the last interglacial (e.g. [38]) and some recently-obtained strontium and U-series isotope dates which place one of the marine calcarenites in the Early Pleistocene (1.73–0.8 mya), and also suggest Middle (0.5–0.4 mya) and Late (70–13 ka) Pleistocene ages for some aeolian calcarenites [34]. The earliest dates are from calcarenites at elevations of 143–165 m and uplift has been attributed to tectonic activity which is still ongoing, as shown by recent earthquakes [27]. However, the wide date and elevation range coupled with a lack of study hinders a precise estimate of the uplift rate and pattern.

Evidence of lower sea level is provided by aeolianite ridges—representing the indurated and calcified remains of Pleistocene sand dunes—sporadically present at the shoreline and offshore. Similar features, usually longitudinal, but sometimes with high domed mounds of 10 m height or more, have been studied elsewhere in the eastern Mediterranean [39–41]. Between Tocra and Ptolemais a largely submerged ridge is visible c. 1–2 km offshore with its location probably reflective of the gentler coastal gradient. At Ptolemais the coast steepens and the foothills of the mountains are just 1.7 km from the beach. Here, the aeolianite ridge constitutes the headland and islands which provided the harbour basins and port buildings for the ancient city. From this point eastwards the indurated dunes repeatedly control the exact format of the shoreline, with scattered offshore islands and broken-through ridges producing repeat patterns of bays, headlands and occasional tombolos. These are particularly clear between Al Oqla and Phycus but are not clearly visible between Phycus and a few kilometres short of Apollonia. At Apollonia itself, a chain of small islands appears offshore and forms the outer boundary of the ancient harbour.

The timing and pattern of Holocene RSL rise from the most recent lowstand is not well studied in Eastern Libya. This contrasts to other Mediterranean regions–exemplified by the Western Mediterranean extending into Tunisia and western Libya [42,43], and Israel [44,45] –which have extensive databases of geological and archaeological Sea Level Index Points (SLIPs) and limiting dates. Consequently, in the study area late Holocene (within the last 2000 years) RSL change has been largely inferred from archaeological evidence, principally the now-submerged ancient harbours of Ptolemais and Apollonia. At Ptolemais, the submergence of structures formerly on dry land, such as quarries, the top of a slipway and a quay have been used to infer an RSL rise of 2–2.5 m [46]. Similarly, at Apollonia, submerged archaeological structures such as slipway bases, quays, a Roman fishtank (Piscina) and *Lithophaga* borings indicate RSL rise of up to 3.5 m [47–49]. Submergence is also evident from other, less well-studied sites. Phycus, Al-Oqla and Al Haniya all have rock-cut features possibly related to fish salting/processing or quarrying which now lie underwater or within the intertidal zone [5,10]. Calculation of functional elevations for, and precise measurement of, these features has yet to be done, but rough estimates suggest that Phycus was submerged by 1–1.5 m, Brak Nota by 2 m and Al-Oqla by 1–2 m [4].

This magnitude of RSL rise exceeds that suggested by the combined effects of late Holocene global eustatic change (fluctuations of 7–11 cm amplitude since 2500 BP: [50]) and regional glacio-isostatic adjustment (10–20 cm RSL rise in the last 2000 years for this coastline: [51]). It also contrasts with reconstructions for western Libya and Tunisia which show only 0.2–0.6m RSL rise within the last ~2000 years driven mainly by glacio-hydro-isostasy and with variations reduced by regional tectonic stability [42,43]. Therefore, the metre-scale RSL rise evidenced at Apollonia and Ptolemais has been attributed to earthquake-related tectonic subsidence. The precise candidate is not yet known. Historical and archaeological data suggest that an earthquake struck Cyrenaica in AD 262, with several during the second half of the fourth century (AD 306–310, 361–363, 365) and another at the end of the fourth century [52]. It has been postulated that Ptolemais remained a working port until the fifth to seventh centuries AD and thus a more likely candidate was the AD 796 earthquake which was centred on Crete but caused damage on the Egyptian coast [46]. Moreover, the evidence from Apollonia further highlights that multiple tectonic events movements could have occurred. For instance, an initial uplift of 0.5–1 m resulted in the slipways being abandoned and built over, and reduced the diameter of the original harbour. A subsequent event then caused ~3 m of subsidence which resulted in the submergence of the harbour to its present depth.

### Archaeology

Apollonia was the ancient port of Cyrene, located ~20 km further inland. Founded in the late seventh century BC, it remained a major port linked to Cyrene until the end of the Byzantine period. Thereafter, Apollonia rose in importance and became the capital of *Libya Superior*, while Cyrene declined. As a functioning port, Apollonia had numerous harbour installations, buildings and industrial features linked to maritime activity. It consisted of two basins, an inner (western) basin and an outer (eastern) basin, both protected from the prevailing north-westerly winds by a chain of reefs and small islands. The inner harbour was more sheltered and probably reserved for warships, at least in the Hellenistic period but perhaps also later. Over time, the harbour received many modifications, including the construction of piers, warehouses, ship sheds and towers. Ceramic sherds strewn across the harbour floor suggest that the outer harbour was mainly reserved for merchant ships. The inner and outer basins were linked by a channel, which was later deepened, probably in the early Roman period. A lighthouse was perched on the easternmost edge of what is today the eastern island. The majority of the Hellenistic- and Roman-period port installations are now submerged up to 2 m below present sea level [3,4,53,54].

Ptolemais was established in the seventh century BC as the harbour of Barka, a city located ~30 km further inland. Initially known as the ‘Harbour at Barca’, it became a named city in its own right in 252 BC under the Ptolemaic dynasty of Egypt [55]. Ptolemais was particularly famous in the Roman world for trading the medicinal herb silphium [46]. The city flourished over the centuries and, in the fourth century AD, was made the capital of the new province of *Libya Superior*. In antiquity, the harbour included two offshore islands that were probably connected to the shore by a mole, creating a two-basin layout similar to Apollonia, but less complex. Also like Apollonia, the port and harbour installations are now submerged by RSL rise [3,46,56].

Like Apollonia, Tocra is said to have been founded from Cyrene in the seventh century BC [57,58]. It prospered to become one of the five cities of the *Pentapolis*, next to Apollonia, Cyrene, Ptolemais and Berenice. Its harbour was an artificial construction with two quays running out from the shore. A mole, ~220 m in length, offered some protection from the elements [8,56–58].

These three case studies were chosen for several reasons. Firstly, they are the best-studied sites on this coast, and therefore have the most supporting information. Secondly, all three are known to have experienced, or currently experience, erosion. Finally, they have been previously subject to a remote sensing condition assessment, but which focused on anthropogenic impacts rather than coastal change [59]. The work presented here therefore presents a counterpart which could be used alongside this previous assessment to provide a holistic understanding of threat for each site.

## Materials and methods

### Background to the approach

This study employs the well-established approach of shoreline change assessment. In essence this uses a Geographical Information System (GIS) to digitally emplace a series of cross-shore transects onto a time-series of shorelines which have been identified and digitized from sources such as historic maps, and aerial/satellite imagery [60]. From these, the rate and/or magnitude of shoreline movement can be calculated. This general approach has been extensively used for coastal management or geomorphological studies [61–66] and more recently also for archaeological vulnerability assessments [67–72].

For this study, we adopted a stepped approach by first using shorelines extracted from medium-resolution (30 m spatial resolution) Landsat imagery which covers the key sites and adjacent coastline. Recent studies have demonstrated that although spatial resolution of Landsat imagery is 30 m, the accuracy of shorelines extracted from said imagery is sub-pixel level: ~ 10–15 m [73–75]. Consequently, as demonstrated in [74] it is feasible to identify shoreline movement over a ~30-year period (i.e. coincident with the start of the 30m-resolution Landsat time-series: 1984) from Landsat imagery where shoreline movement rates are sufficiently high (> 0.5 m/yr) to result in an overall shoreline position change that exceeds the aforementioned sub-pixel accuracy of ~15 m. Therefore, in the study area, the Landsat-based approach allowed detection of general trends of coastal change since the mid-1980s. However, the spatial resolution of the imagery is insufficient to detect smaller instances of coastal retreat/erosion which could still adversely impact coastal archaeological sites. To remedy this, as a second step we used Very High Resolution (VHR: <1 m spatial resolution) commercial satellite imagery acquired within the last decade and supplemented, where available, by historic air photos or declassified spy satellite imagery from the 1970s. This enabled detailed characterization of shoreline change in and around the historic core of each site.

### Shoreline extraction: Landsat imagery

Annual composite shorelines for the period 1985–2020 were extracted from Landsat imagery following the method of [74]. Composite shorelines—representing the land-water interface generalized from multiple individual shorelines for a given year—were used for four reasons (see also [73]). Firstly, to smooth out the impact of waves and tides on individual shoreline positions; secondly, to account for variations in individual shoreline position caused by the spatial resolution of the source imagery; thirdly, to exclude cloud cover and, finally, to reduce the amount of shorelines extracted from several hundred Landsat images to a usable number. Detection and extraction steps included (see S1 Supporting Information in S1 File for additional details):

- Using Google Earth Engine (GEE: [76]), Landsat 5, 7 and 8 Surface Reflectance imagery from 1985–2020 were selected and filtered to include only images which:
  ○ cover the study sites
  ○ have good location accuracy (Geometric RMSE <10m)
  ○ low cloud cover (overall image cloud cover <25%)
- Images were composited by year and using a 15^th^ Percentile reducer following [74]. This enabled exclusion of pixels affected by clouds, sun-glint and white water.
- Shorelines were extracted from the composite images using the dynamic thresholding approach of [77]. Only composites created using >5 images were used.
- Extracted shorelines were manually checked and cleaned to remove erroneous values (e.g. false shorelines caused by cloud shadows). A Gaussian smoothing function was employed to reduce their pixelated appearance.

Rate of change statistics were calculated for the extracted shorelines using the Digital Shoreline Analysis System (DSAS) 5.1 add-in for ArcGIS 10 [78]. The similar, but global-scale, approach of [74] employed cross-shore transects at 500 m spacing. However, we used closer-spaced transects to enable more detailed assessment of shoreline change [79]. Key DSAS parameters were:

- 50 m transect spacing.
- Fixed uncertainty value of ±15 m based on the accuracy of detected shorelines [74]
- Calculated Linear Regression Rate (LRR) LRR gives the rate of shoreline change based on linear regression applied to all shoreline positions in the time series.
- Calculated 90% confidence interval. The confidence interval measures the uncertainty of the calculated rate based on user-supplied uncertainty values. Applying this to the LRR effectively allows filtering out of apparent shoreline movements which are actually a product of image pixel resolution, or other factors such as geo-referencing or digitization errors.

### Shoreline extraction: VHR imagery

For each site we used four orthorectified and co-registered VHR images which were acquired within the last 10–12 years at intervals of 2–4 years (Table 1). VHR imagery was obtained in georeferenced but non-orthorectified form (OR2A-level). Orthorectification was performed in ArcGIS using the provided satellite Rational Polynomial Coefficients (RPC) and the SRTM (30 m) digital elevation model (DEM). For each site, the image with the smallest nadir angle was chosen as the reference image. The other satellite orthoimages were then co-registered to it using common visible features. Extension of this recent time series back by ~50 years was enabled by declassified spy satellite (KH-9) imagery dating to 1974 and which was available for all sites. For Apollonia, this was pushed back even further through use of scanned aerial photographs dating to 1949. In all cases, common visible features were used to co-register the historic imagery to the reference VHR image.

**Table 1:** List of VHR images and associated metadata per study site.

Site	Sensor/Source	Image Type	Date	Spatial resolution (m)	Co-registration RMSE (m)
Apollonia	GeoEye-1/Maxar^a^	4-band multispectral	18/10/2010	0.5	N/A: Reference image
Apollonia	GeoEye-1/Maxar^a^	4-band multispectral	22/06/2014	0.5	0.47
Apollonia	Worldview-2/Maxar^a^	4-band multispectral	08/11/2017	0.5	0.69
Apollonia	GeoEye-1/Maxar^a^	4-band multispectral	07/11/2019	0.5	0.6
Apollonia	KH-9/USGS^b^	Black & white	03/02/1974	~0.6–1.2	0.66
Apollonia	Aerial frame camera/Hunting Survey^c^	Black & white	-/-/1949	~0.5	3.08
Apollonia	Aerial frame camera/Hunting Survey^c^	Black & white	-/-/1949	~0.5	1.9
Apollonia	Aerial frame camera/Hunting Survey^c^	Black & white	-/-/1949	~0.5	1.41
Apollonia	Aerial frame camera/Hunting Survey^c^	Black & white	-/-/1949	~0.5	1.66
Apollonia	Aerial frame camera/Hunting Survey^c^	Black & white	-/-/1949	~0.5	2.36
Apollonia	Aerial frame camera/Hunting Survey^c^	Black & white	-/-/1949	~0.5	3.19
Tocra	GeoEye-1/Maxar^a^	4-band multispectral	26/05/2009	0.5	0.36
Tocra	Worldview-3/Maxar^a^	4-band multispectral	11/11/2014	0.5	0.35
Tocra	Worldview-3/Maxar^a^	4-band multispectral	06/10/2017	0.5	N/A: Reference image
Tocra	Worldview-3/Maxar^a^	4-band multispectral	21/03/2020	0.5	0.42
Tocra	KH-9/USGS^b^	Black & white	03/02/1974	~0.6–1.2	0.74
Ptolemais	GeoEye-1/Maxar^a^	4-band multispectral	29/03/2009	0.5	N/A: Reference image
Ptolemais	Worldview-2/Maxar^a^	4-band multispectral	24/08/2012	0.5	0.73
Ptolemais	Worldview-3/Maxar^a^	4-band multispectral	11/11/2014	0.5	0.39
Ptolemais	Worldview-2/Maxar^a^	4-band multispectral	25/04/2016	0.5	0.28
Ptolemais	KH-9/USGS^b^	Black & white	03/02/1974	~0.6–1.2	1.39

^*a*^Data provided by the European Space Agency (ESA) under the Third Party Mission Program.

^*b*^Data provided by the United States Geological Survey [80].

^*c*^Data provided by the British Institute for Libyan and North Africa Studies.

Shoreline proxies were then identified from the images and manually digitised at scales of 1:300–1:600. For Apollonia and Tocra, these were eroding clifflines, scarps or breaks in slope. It is these types of locations where destructive erosion exposes buried archaeological remains, or causes collapse of structures built on the clifftop. For Ptolemais, consistent backshore scarps were not present across all images. Therefore, the instantaneous land-water boundary (aka the waterline) was also used as the primary shoreline proxy. Rate of change statistics were again calculated using DSAS 5.1 with the following parameters:

- 10 m (Apollonia, Tocra) or 20 m (Ptolemais) transect spacing.
- Variable uncertainty incorporating:
  ○ Co-registration error (RMSE); varies per image (Table 1).
  ○ Image spatial resolution; varies per image (Table 1).
  ○ Constant digitization error of 0.75m found by manually digitizing the same proxy three times and finding the average distance between digitized shorelines.

For each site, calculations were performed for three imagery subsets:

1. All VHR images: time series from the earliest to the latest image.
2. Recent VHR images only: time series covering the last decade.
3. Earliest historic image and earliest recent VHR image: time series covering the period prior to the last decade.

For all subsets, the End Point Rate (EPR) of shoreline change was calculated. This takes only two digitized shorelines (the earliest and latest) and calculates the rate of change required to move from the former to the latter. The LRR was also calculated but only for the first and second subset because it requires at least three shorelines. As for the Landsat analysis (see above), a 90% confidence interval was also calculated in order to enable exclusion of transects whose apparent rates of change could be caused by errors in digitization, co-registration or image resolution.

### Forecasting future change

Future shoreline positions were estimated using the beta shoreline forecasting tool which is built into DSAS 5.1 [79]. This tool uses the calculated LRR combined with a Kalman filter to project a shoreline position forward in time as well as providing a measure of uncertainty for said position (see [78] for details of the tool’s operation). Given the complexity of processes driving shoreline change, this tool only provides forecasts for 10 and 20 years beyond the analysis date (i.e. 2032 and 2042 in this case). Analogous studies have been completed elsewhere for coastal management purposes [81–86] and for cultural heritage vulnerability assessment [87]. For this study, the forecasting tool was applied only to the LRR derived from VHR shorelines for the last decade. This was done to ensure that projections were based on the most recent observed patterns of shoreline movement. To assess future impacts on each archaeological site, the positions of the forecast shorelines were then manually compared against the location of known archaeological material and structures.

## Results

### Shoreline change: Landsat

#### Apollonia

**Fig 2:**
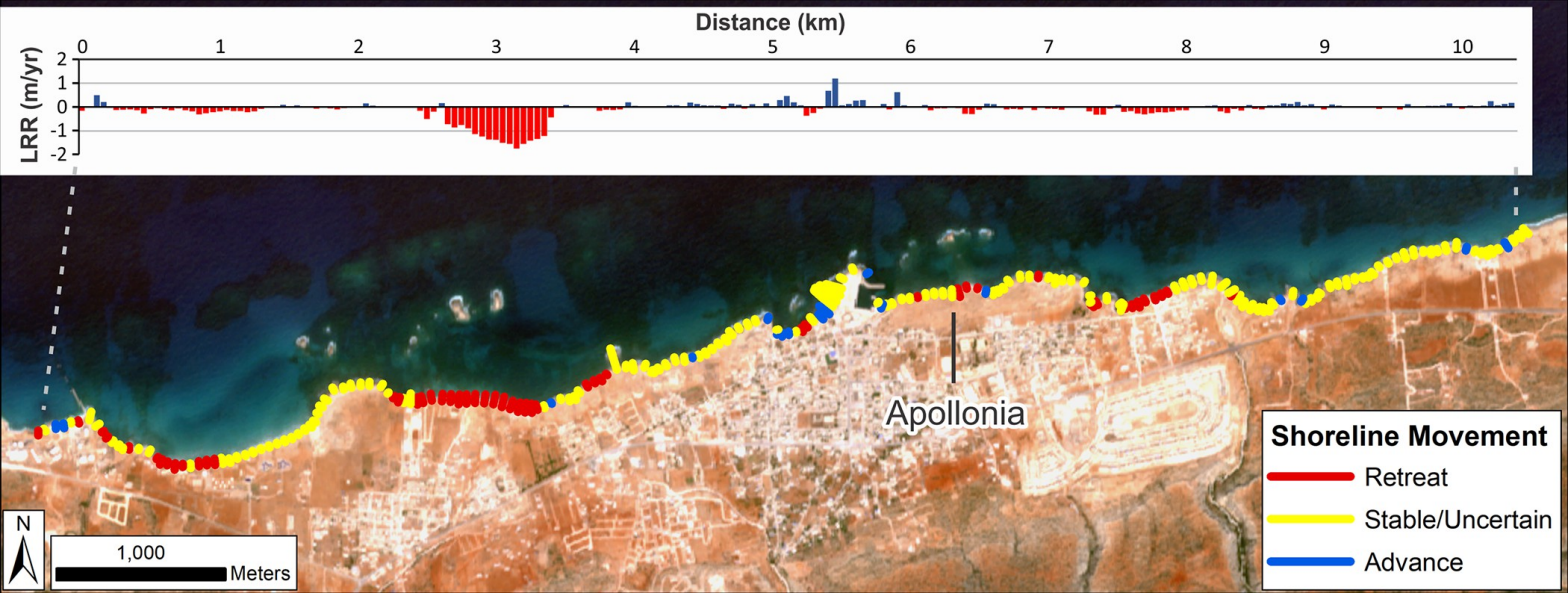
Shoreline change for the wider Apollonia area (Landsat: 1985–2020). DSAS shoreline change transects classified into statistically significant categories based on LRR and 90% LCI as obtained from Landsat imagery. Quantified LRR (metres/year) is plotted on the inset graph with negative values (red) indicating erosion/retreat and positive values (blue) indicating accretion/advance. Label Apollonia indicates the ancient site. Basemap: Sentinel-2 (from the Copernicus Program; 2020 annual composite created using GEE).

Roughly 56% of measured transects have negative LRR values suggestive of coastal retreat (Table 2; Fig 2). However, the caveat is that many transects have a low LRR value (±0.5 m/yr). Therefore, considering the spatial resolution of the input Landsat imagery, it is uncertain if these low values definitely show trends of shoreline movement. Applying the 90% confidence intervals, only 25% of transects show a statistically significant trend of retreat. Even fewer transects (8.2%) show statistically significant accretion. Thus, most of this study area is characterized by stability or low magnitude change which cannot be definitely classified as erosion or accretion. Even so, distinct loci of change are identifiable. The largest cluster of erosional transects is located ~2–3 km west of the ancient site with smaller clusters ~4 km to its west and 500 m to its east. At the ancient site itself, retreat transects are located on the shoreline immediately east of the modern harbour.

**Table 2:** Summary DSAS results based on Landsat imagery for wider areas around Apollonia, Ptolemais and Tocra.

	Apollonia (Area)	Ptolemais (Area)	Tocra (Area)
**Total number transects**	208	338	294
**Average LRR (m/yr)**	-0.11	-0.91	-0.04
**Average LCI (m/yr)**	0.17	0.21	0.18
**Erosional transects: %**	56.3%	92.6%	43.2%
**Statistically significant erosional transects: %**	25.2%	79%	24.8%
**Max erosion rate (m/yr)**	-1.75	-2.48	-1.75
**Average erosion rate (m/yr)**	-0.27	-0.99	-0.32
**Accretional transects: %**	43.8%	7.5%	56.8%
**Statistically significant accretional transects: %**	8.2%	0.89%	29.9%
**Max accretion rate (m/yr)**	1.19	0.31	0.6
**Average accretion rate (m/yr)**	0.11	0.08	0.17

LRR, Linear Regression Rate (for all transects); LCI, Confidence interval of linear regression (90%); erosional transects are those with negative LRR; accretional transects are those with positive LRR; statistically significant erosional/accretional transects are those which still have negative/positive LRR after inclusion of LCI.

#### Ptolemais

**Fig 3:**
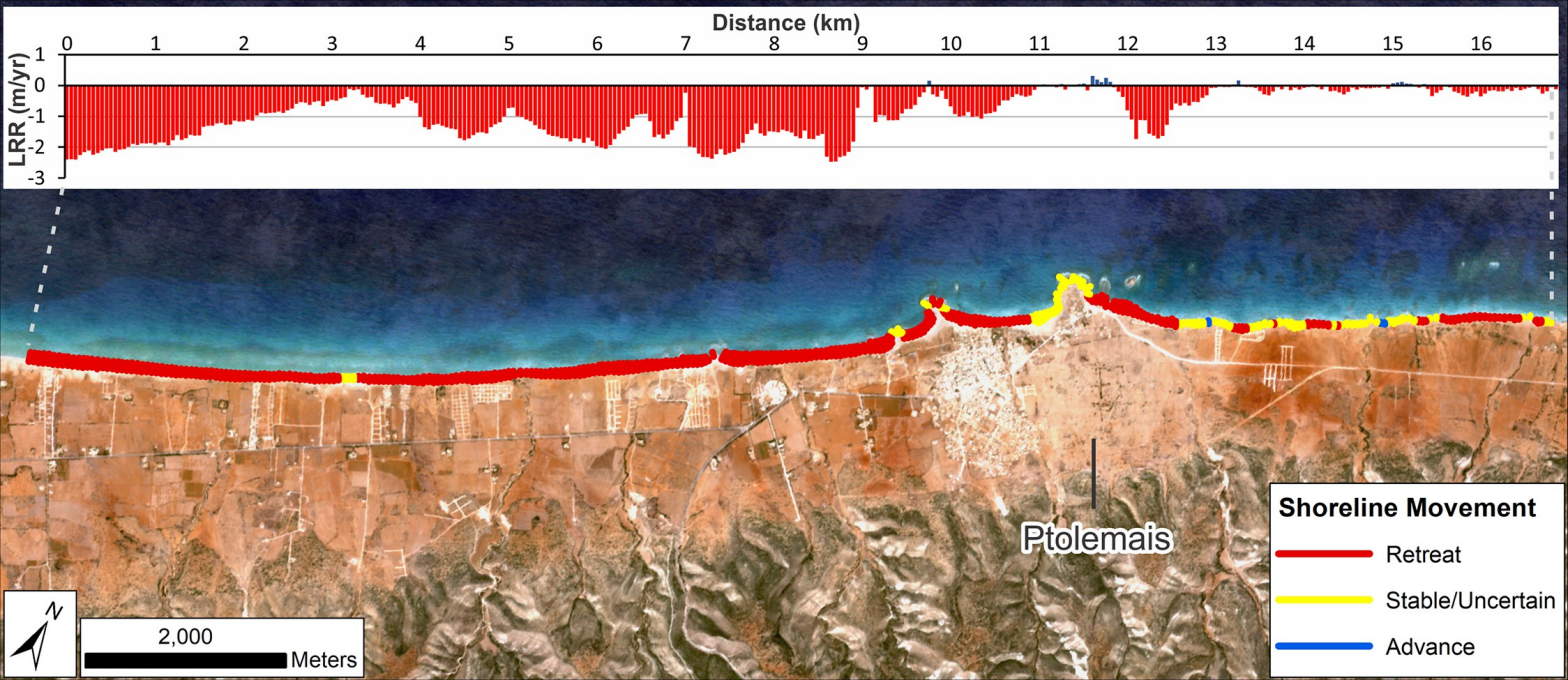
Shoreline change for the wider Ptolemais area (Landsat: 1985–2020). DSAS shoreline change transects classified into statistically significant categories based on LRR and 90% LCI as obtained from Landsat imagery. Quantified LRR (metres/year) is plotted on the inset graph with negative values (red) indicating erosion/retreat and positive values (blue) indicating accretion/advance. Label Ptolemais indicates the ancient site. Basemap: Sentinel-2 (from the Copernicus Program; 2020 annual composite created using GEE).

This area shows a very strong tendency towards erosion: 93% of transects have negative LRR values (Table 2). Even when confidence intervals are included, 79% of transects still show statistically significant erosion/retreat (Table 2, Fig 3). Considering the resolution of the Landsat imagery, this is a strong indication that coastal erosion and retreat have been extensive and rapid over the last 35 years. Only areas on the headlands and to the northeast of the ancient site appear to be unaffected or minimally impacted.

#### Tocra

**Fig 4:**
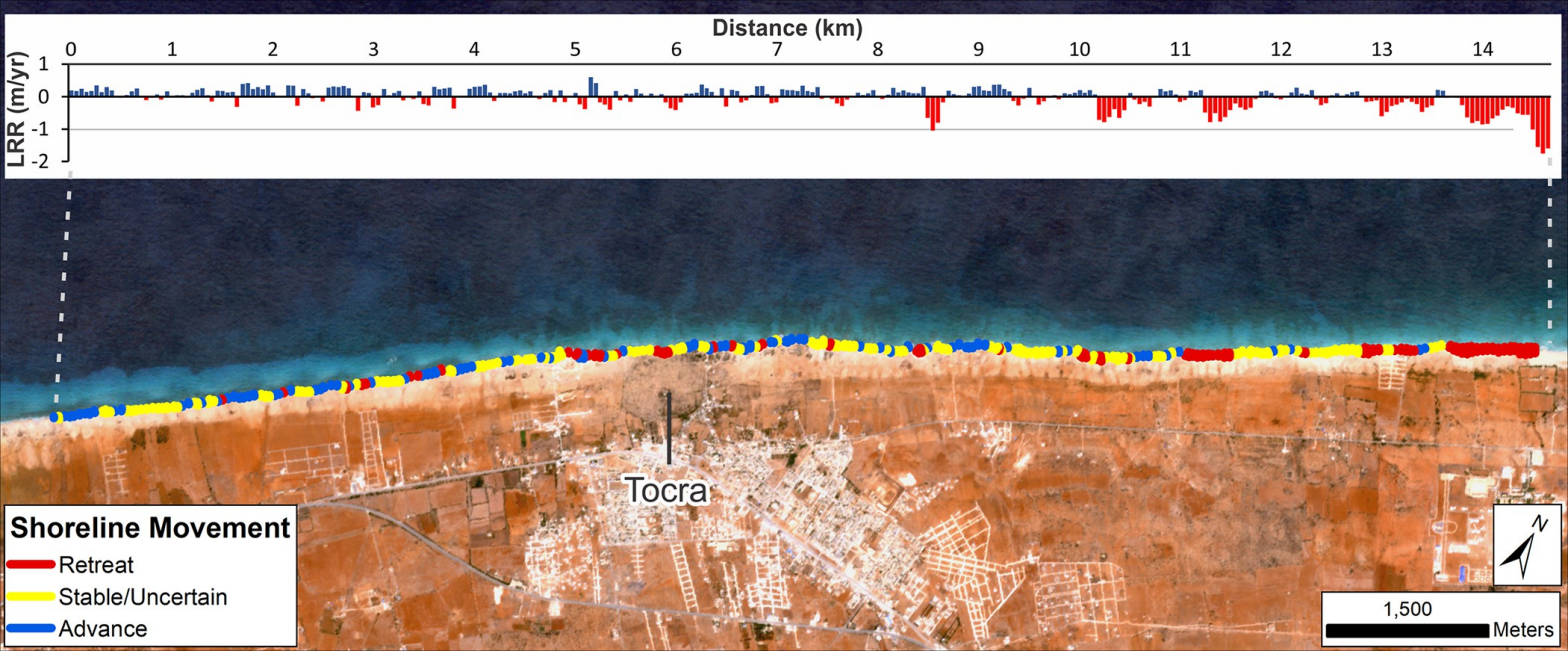
Shoreline change for the wider Tocra area (Landsat: 1985–2020). DSAS shoreline change transects classified into statistically significant categories based on LRR and 90% LCI as obtained from Landsat imagery. Quantified LRR (metres/year) is plotted on the inset graph with negative values (red) indicating erosion/retreat and positive values (blue) indicating accretion/advance. Label Tocra indicates the ancient site. Basemap: Sentinel-2 (from the Copernicus Program; 2020 annual composite created using GEE).

Measured shoreline change rates around Tocra suggest a roughly even balance between accretion/advance and erosion/retreat (~57% and 43% of transects respectively). Inclusion of confidence intervals causes these percentages to fall to ~30% and ~25% for erosion and accretion respectively (Table 1; Fig 4). When mapped spatially, these suggest that the general area is characterised by a mix of slow advance and retreat. There is also a suggestion of reduced erosion southwest of the archaeological site which increases moving northeast along the coastline. This latter area marks the start of the extensive stretch of eroding shore identified to the southwest of Ptolemais. Importantly, even when confidence intervals are included, there still appear to be distinct patches of erosion in the centre of the ancient site and immediately to its southwest.

### Shoreline change: VHR imagery

#### Apollonia

**Fig 5:**
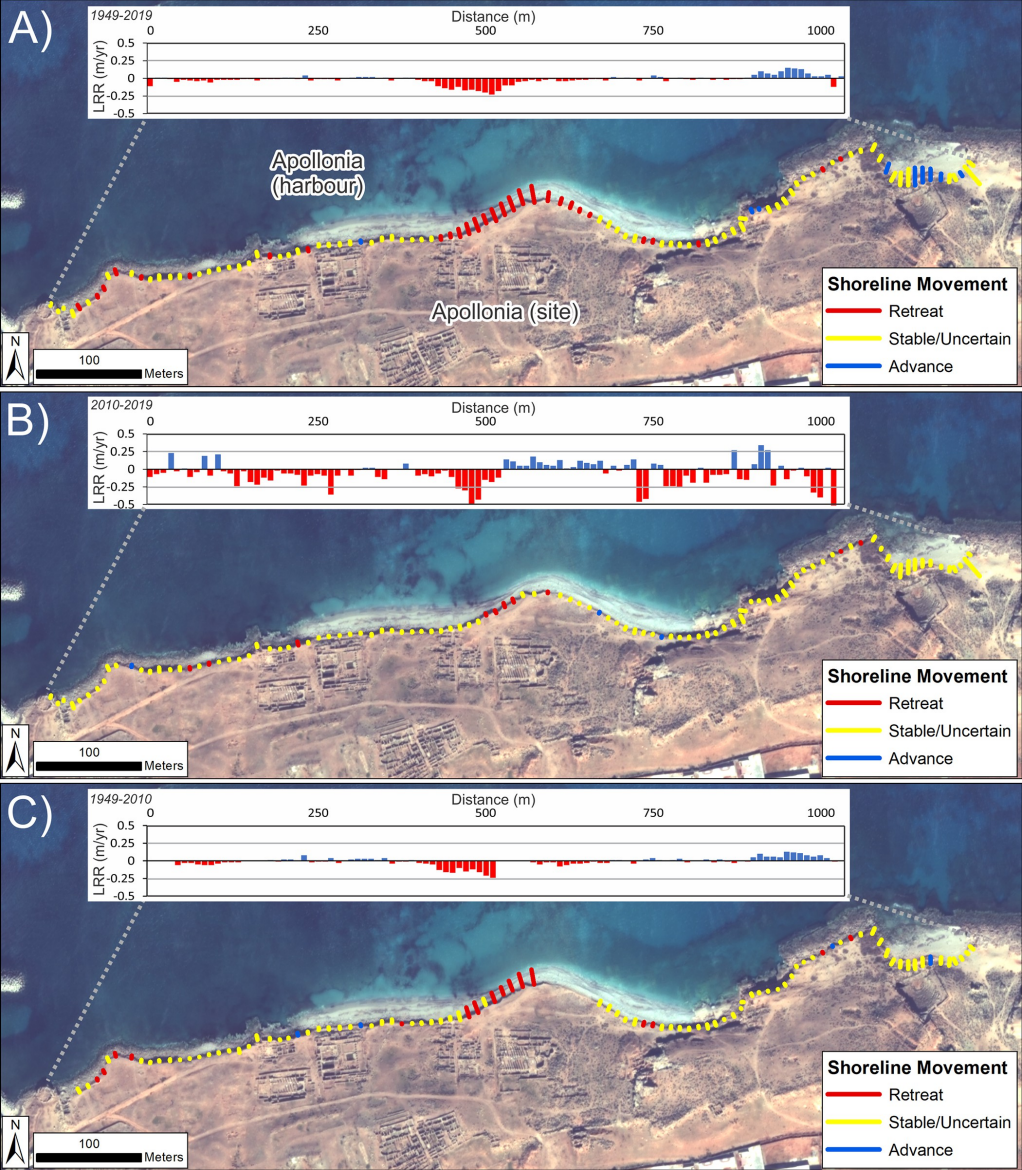
Shoreline change for the Apollonia (VHR images). DSAS shoreline change transects classified into statistically significant categories based on LRR and 90% LCI as obtained from VHR imagery. Quantified LRR (metres/year) is plotted on the inset graph with negative values (red) indicating erosion/retreat and positive values (blue) indicating accretion/advance. Shoreline proxy is the backshore cliff. A) Full times series: 1949–2019; B) Recent: 2010–2019 and C) Historic to recent: 1949–2010. Basemap: ©Maxar (7/11/2019), provided by European Space Imaging.

Analysis focussed on the backshore scarp/cliffline at the ancient site where eroding archaeological material has been observed. Calculated rates of change are summarized in Table 3 and mapped in Fig 5. These suggest that, taking into account the full shoreline times series (1949–2019) and including the 90% confidence intervals, definite erosion has been relatively limited (Table 3, Fig 5A). The clearest location is on the western side of the bulge in the coastline in the centre of the site, a feature which could be an incipient tombolo. In addition, isolated spots of erosion occur to the west and east of this feature. Overall erosion rates have been relatively low, <-0.25 m/yr, (LRR values).

**Table 3:** Summary DSAS results based on VHR imagery for Apollonia.

	1949–2019	2010–2019	1949–2010
End Point Rate (EPR)			
**Total number transects**	104	104	103
**Average EPR (m/yr)**	-0.02	-0.04	-0.02
**Average ECI**	0.05	0.23	0.05
**EPR Erosional transects: %**	64.4	60.6	60.2
**EPR Statistically significant erosional transects: %**	17.3	11.5	17.5
**EPR Max erosion rate (m/yr)**	-0.22	-1.02	-0.24
**EPR Average erosion rate (m/yr)**	-0.05	-0.15	-0.05
**EPR Accretional transects: %**	35.6	39.4	39.81
**EPR Statistically significant accretional transects: %**	8.7	3.9	10.7
**EPR Max accretion rate (m/yr)**	0.12	0.3	0.13
**EPR Average accretion rate (m/yr)**	0.03	0.12	0.04
**Linear Regression Rate (LRR)**			
**Average LRR rate (m/yr)**	-0.02	-0.07	-0.01
**Average LCI (m/yr)**	0.04	0.48	0.14
**LRR Erosional transects** ^**g**^ **: %**	65.4	64.4	59
**LRR Statistically significant erosional transects: %**	32.7	9.6	17.9
**LRR Max rate (m/yr)**	-0.23	-1.72	-0.24
**LRR Average erosion rate (m/yr)**	-0.05	-0.17	-0.05
**LRR Accretional transects: %**	34.6	35.6	41.1
**LRR Statistically significant accretional transects: %**	11.5	2.9	5.3
**LRR Max accretion (m/yr)**	0.15	0.34	0.12
**LRR Average accretion rate (m/yr)**	0.04	0.1	0.04

ECI: Confidence interval of EPR (90%), LCI, Confidence interval of linear regression (90%); erosional transects are those with negative LRR/EPR; accretional transects are those with positive LRR/EPR; statistically significant erosional/accretional transects are those which still have negative/positive LRR /EPR after inclusion of LCI/ECI.

A different pattern emerges when the time series is split into pre- and post-2010 subsets. Between the western-most edge of the study area and the incipient tombolo, a long-term trend (1949–2010) of very slow erosion or stability may have switched to a slow rate of retreat in the last 10 years. This is suggested by the higher rates of retreat observed in the past decade (Fig 5B and 5C). The caveat is that the majority of transects fall into the uncertain category if 90% confidence intervals are included. Nevertheless, this still means that erosion cannot be ruled out.

**Fig 6:**
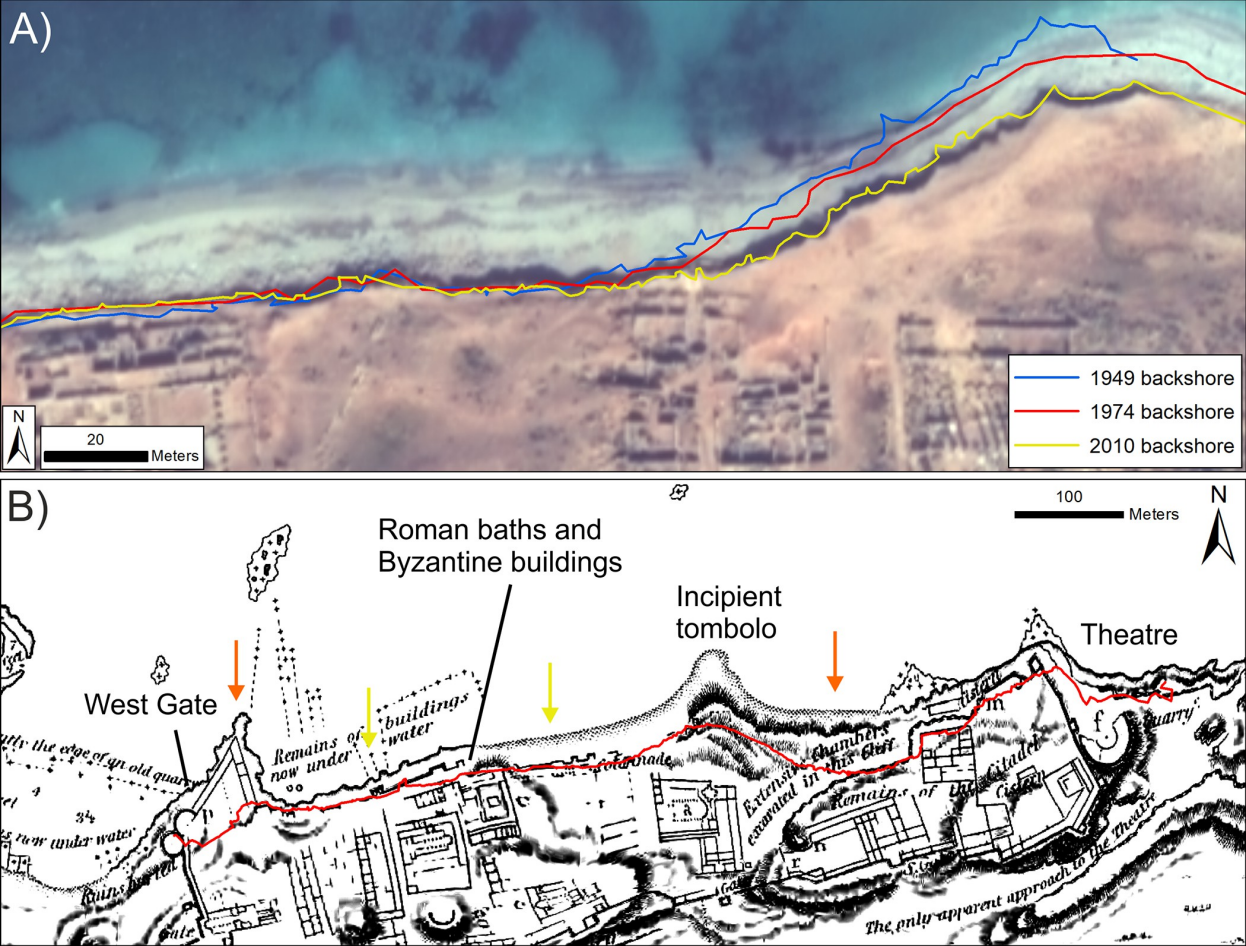
Erosion examples at Apollonia from historic map and VHR imagery. A) Close-up of the incipient tombolo’s western side and adjacent shoreline (VHR image: ©Maxar [7/11/2019], provided by European Space Imaging). Earlier positions of the eroding backshore scarp are superimposed. Note difference in former scarp positions on the tombolo versus the more stable coastline to the west. B) Excerpt from the Beecheys’ map of Apollonia [6], georeferenced and co-registered to a recent VHR satellite image. Red line marks location of the backshore scarp from the 2019 VHR image. Orange and yellow arrows respectively mark locations where extensive and minor coastal change are suggested by the VHR image–historic map comparison.

Faster retreat, up to -0.5 m/yr, characterises the western side of the incipient tombolo for the last decade. This is faster than that for the period 1949–2009 (<-0.25 m/yr). Erosion here is definitive even when confidence intervals are included and can be clearly observed on the recent VHR images (Fig 6A). In contrast, the eastern side of the tombolo appears to have been relatively stable over the past decade (Fig 5B), though it may have experienced erosion in the past (Fig 5C).

East of the incipient tombolo, the slopes above the rocky foreshore appear to have been relatively stable over the long term, but now appear to be retreating more rapidly (Fig 5B and 5C). Finally, in the eastern-most part of the study area, the situation is less clear. There appears to be a long-term trend of stability or advance which has given way to more variability including both advance and retreat. Again, the majority of transects fall within the uncertain category. The imagery suggests that this complex pattern may be due to movement of a beach berm rather than erosion.

In short, these data could be interpreted to show that erosion is present and has accelerated with the last decade. A long-term trend of slow erosion is supported by comparison with 19^th^ century accounts. Apollonia was mapped by the Beechey brothers during their 1821–22 survey of the Libyan coast. They noted that “*portions of the elevated ground on which the front of the town has been built are continually falling in from this cause* [coastal erosion]” ([6]: pg. 572). Aside from construction of the modern harbour, their map showed some areas of difference which could be the result of coastal erosion/retreat (Fig 6B):

1. The loss of a peninsula and associated structures which extended from the West Gate Tower.
2. The possible loss of walls or structures seaward of the Roman baths and a Byzantine industrial building.
3. A narrowing and reduction in length of the incipient tombolo. The map also hints at ruins extending into the tombolo and which are not presently visible.
4. A coastal scarp extending further seaward on the eastern side of the tombolo.

Thus, despite the long-term trend of erosion, it has not affected the entirety of the ancient site. If observed late 20^th^ century erosion rates of -0.2 m/yr had prevailed over the 200 years since the Beecheys’ visit, the coastline would have retreated by 40 m. This level of change is not apparent from a comparison of the Beecheys’ map and the recent VHR images with the exception of the structures extending from the West Gate tower, the tip of the tombolo and possible the coastal scarp immediately to its east (Fig 6).

#### Ptolemais

**Fig 7:**
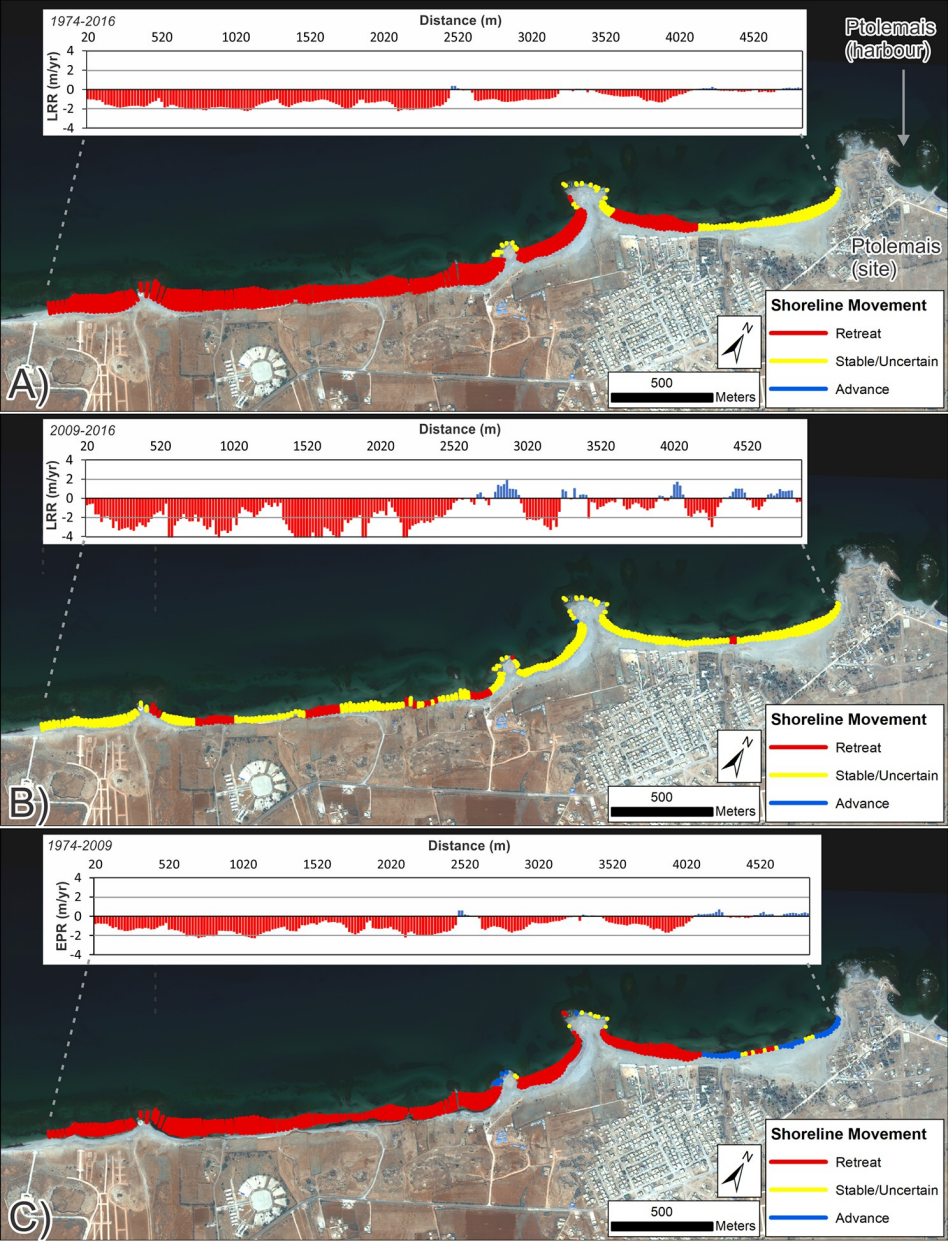
Shoreline change for southwest of Ptolemais (VHR images). DSAS shoreline change transects classified into statistically significant categories based on LRR and 90% LCI, or EPR and 90% ECI as obtained from VHR imagery. Quantified LRR or EPR (metres/year) is plotted on the inset graph with negative values (red) indicating erosion/retreat and positive values (blue) indicating accretion/advance. Shoreline proxy is the waterline. A) Full times series: 1974–2016; B) Recent: 2009–2016 and C) Historic to recent: 1974–2009. Basemap: ©Maxar (25/04/2016), provided by European Space Imaging.

**Fig 8:**
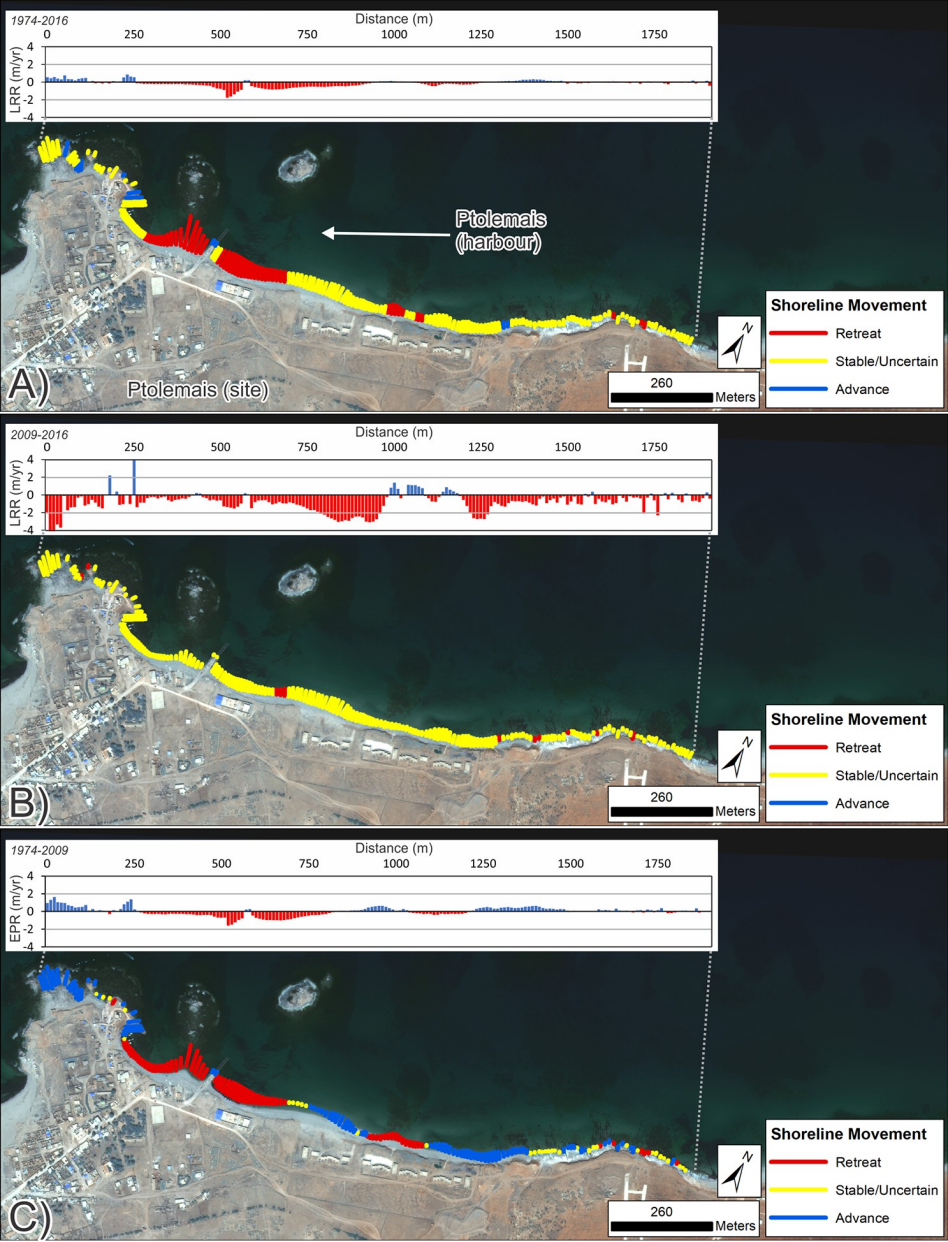
Shoreline change for Ptolemais headland and to the northeast (VHR images). DSAS shoreline change transects classified into statistically significant categories based on LRR and 90% LCI, or EPR and 90% ECI as obtained from VHR imagery. Quantified LRR or EPR (metres/year) is plotted on the inset graph with negative values (red) indicating erosion/retreat and positive values (blue) indicating accretion/advance. Shoreline proxy is the waterline. A) Full times series: 1974–2016; B) Recent: 2009–2016 and C) Historic to recent: 1974–2009. Basemap: ©Maxar (25/04/2016), provided by European Space Imaging.

Analysis focussed on the ancient site plus the coast extending to the southwest. This extended area was examined because the Landsat-derived shoreline change assessment showed major coastal retreat to the southwest. Calculated rates of change are summarized in Tables 4 and 5 and mapped in Figs 7 and 8.

**Table 4:** Summary DSAS results based on VHR imagery for southwest of Ptolemais headland.

	1974–2016	2009–2016	1974–2009
**End Point Rate (EPR)**			
**Total number transects**	242	269	240
**Average EPR (m/yr)**	-1	-1.4	-1
**Average EPR confidence interval**	0.08	0.32	0.09
**EPR Erosional transects: %**	85.1	78.1	82.5
**EPR Statistically significant erosional transects: %**	80.2	71.4	80
**EPR Max erosion rate (m/yr)**	-2.2	-10.7	-2.3
**EPR Average erosion rate (m/yr)**	-1.2	-2.2	-1.2
**EPR Accretional transects: %**	14.9	21.9	17.5
**EPR Statistically significant accretional transects: %**	8.3	17.5	12.9
**EPR Max accretion rate (m/yr)**	0.6	7.4	0.7
**EPR Average accretion rate (m/yr)**	0.2	1.4	0.2
**Linear Regression Rate (LRR)**			
**Average LRR rate (m/yr)**	-1.1	-1.5	-
**Average LCI (m/yr)**	0.6	4.8	-
**LRR Erosional transects: %**	89.2	81.4	-
**LRR Statistically significant erosional transects: %**	73.9	14.9	-
**LRR Max rate (m/yr)**	-2.2	-5.4	-
**LRR Average erosion rate (m/yr)**	-1.2	-2	-
**LRR Accretional transects: %**	10.8	18.6	-
**LRR Statistically significant accretional transects: %**	0	0.4	-
**LRR Max accretion (m/yr)**	0.4	1.9	-
**LRR Average accretion rate (m/yr)**	0.1	0.7	-

ECI: Confidence interval of EPR (90%), LCI, Confidence interval of linear regression (90%); erosional transects are those with negative LRR/EPR; accretional transects are those with positive LRR/EPR; statistically significant erosional/accretional transects are those which still have negative/positive LRR /EPR after inclusion of LCI/ECI.

**Table 5:** Summary DSAS results based on VHR imagery for Ptolemais headland and to the northeast.

	1974–2016		2009–2016		1974–2009	
	Waterline	Backshore	Waterline	Backshore	Waterline	Backshore
**End Point Rate (EPR)**						
**Total number transects**	192	184	192	184	191	165
**Average EPR (m/r)**	-0.14	-0.04	-0.8	0.1	-0.01	0.02
**Average EPR confidence interval**	0.08	0.1	0.3	0.3	0.09	0.09
**EPR Erosional transects: %**	63	39.7	80.7	37.5	45.5	48.5
**EPR Statistically significant erosional transects: %**	47.9	11.4	71.3	18.5	40.3	6.7
**EPR Max erosion rate (m/yr)**	-1.6	-2.7	-4.4	-2.7	-1.6	-0.4
**EPR Average erosion rate (m/yr)**	-0.4	-0.3	-1.2	-0.6	-0.4	-0.07
**EPR Accretional transects: %**	37	60.3	19.3	62.5	54.5	51.5
**EPR Statistically significant accretional transects: %**	30.2	21.7	11.5	28.8	41.4	19.4
**EPR Max accretion rate (m/yr)**	1.4	1.1	7.19	7	1.6	0.63
**EPR Average accretion rate (m/yr)**	0.3	0.1	0.85	0.5	0.3	0.1
**Linear Regression Rate (LRR)**						
**Average LRR rate (m/yr)**	-0.15	-0.06	-0.9	-0.01	-	-
**Average LCI (m/yr)**	0.44	0.4	3.5	1.4	-	-
**LRR Erosional transects: %**	65.6	47.8	83.9	42.9	-	-
**LRR Statistically significant erosional transects: %**	25.5	3.3	5.7	3.3	-	-
**LRR Max rate (m/yr)**	-1.8	2.9	-5.4	-2.9	-	-
**LRR Average erosion rate (m/yr)**	-0.3	-0.2	-1.2	-0.6	-	-
**LRR Accretional transects: %**	34.4	52.2	16.2	57.1	-	-
**LRR Statistically significant accretional transects: %**	5.7	13	0	5.4	-	-
**LRR Max accretion (m/yr)**	0.9	0.6	5.2	5.4	-	-
**LRR Average accretion rate (m/yr)**	0.2	0.1	0.7	0.4	-	-

ECI: Confidence interval of EPR (90%), LCI, Confidence interval of linear regression (90%); erosional transects are those with negative LRR/EPR; accretional transects are those with positive LRR/EPR; statistically significant erosional/accretional transects are those which still have negative/positive LRR /EPR after inclusion of LCI/ECI.

To the southwest, assessment used the instantaneous waterline as a shoreline proxy because the eroding cliffline visible in recent images is not apparent on the 1974 satellite images; it is evidently a feature created by post-1974 erosion. Therefore, the waterline represented the only consistent shoreline proxy visible in all images. The caveat is that uncertainty increases because its position can change with waves and tides as well as longer-term shoreline advance/retreat. For the area to the northwest, rates of change were calculated using both the waterline and the backshore scarp.

Results confirm the strong pattern of coastal erosion/retreat previously identified from Landsat. The VHR images provide further detail and show clear difference between rocky headlands—largely stable over the period of observation—and beaches, which have almost universally retreated (Fig 7A). The exception is the beach forming the western side of the Ptolemais headland. This has been largely stable, possibly even advancing slightly in places. Note though that for the recent period (Table 4; Fig 7B), large LRR confidence intervals cause many transects to be classified as uncertain. In part, this could be attributed to movement of the instantaneous waterline caused by waves/tides. Nevertheless, even if this is accounted for, there are still clear zones of definitive beach retreat even over this relatively short (7-year) interval.

These data also suggest that rates of erosion have increased. Using the EPR from 1974–2009, rates of retreat average ~-1 m/yr. The equivalent for the last decade is ~-1.4 m/yr (Fig 7B and 7C; Table 5). The same increase is true of maximum values. While some of these extreme rates are likely the product of variation caused by waves/tides, the overall evidence of rapid recent retreat is very clear. The reduction, and in some locations, near-total loss of the beach has allowed waves to reach the backshore and cut the vertical cliff that now lines most of the western part of the study area and which did not exist in 1974 (Fig 7). With regard to the cliffline itself, it is apparent that some locations have retreated by up to 15–17m between 2009–2016.

**Fig 9:**
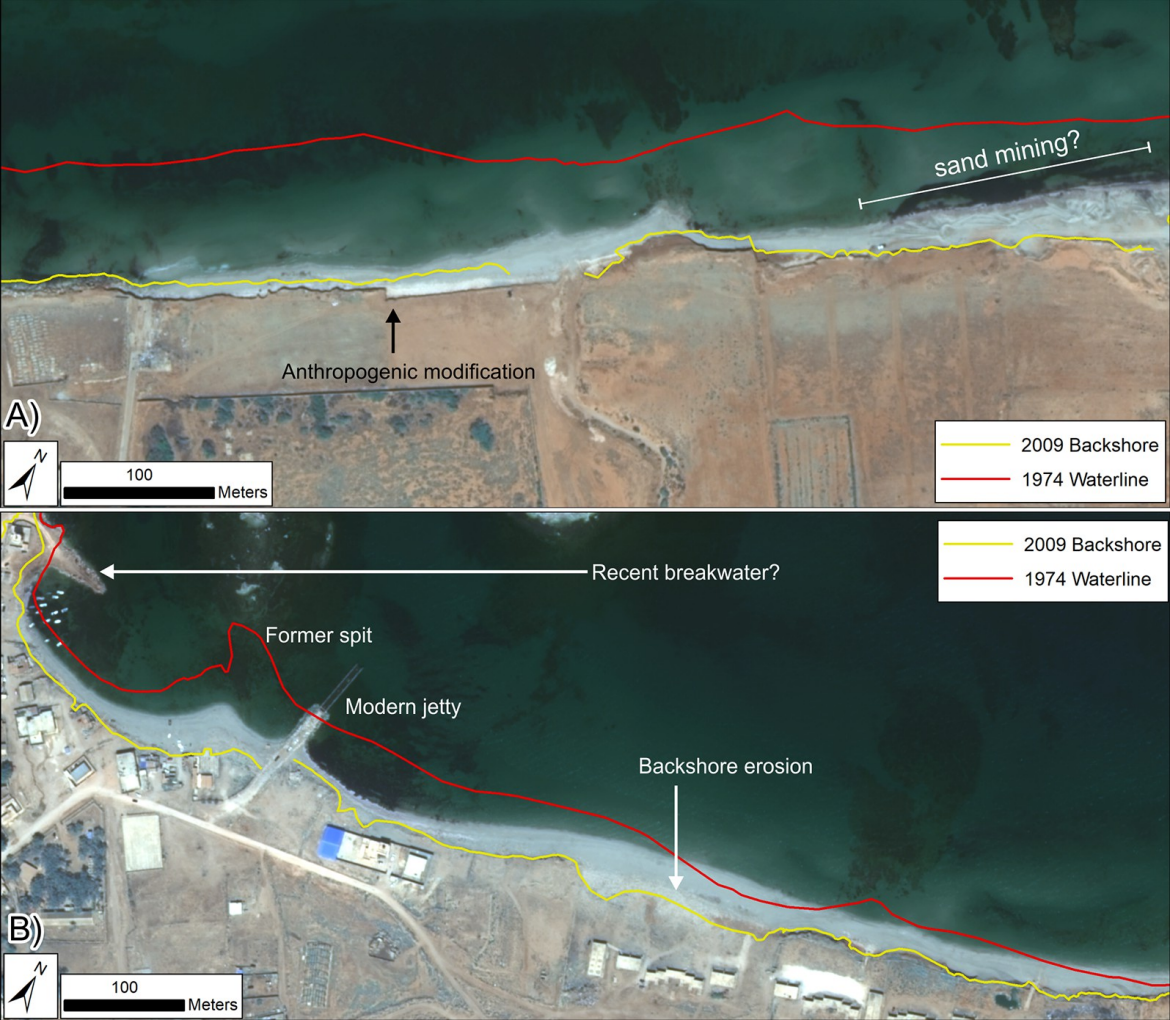
Examples of coastal change around Ptolemais from VHR images. For both examples the digitized 2009 backshore cliff/scarp (yellow) and 1974 waterline (red) are superimposed onto the 2016 VHR image (©Maxar (25/04/2016), provided by European Space Imaging). A) Southwest of Ptolemais showing extensive beach and cliffline retreat. Arrow indicates an unnaturally straight cliffline suggestive of human modification. Also shown are possible traces of recent sand mining on the beach. B) Northeast of Ptolemais showing loss of a former spit, recent structures built around the ancient harbour and possible backshore erosion.

Around the ancient harbour itself, there is strong evidence of waterline retreat between 1974–2009 (Figs 8C and 9B). Most of this occurred in the vicinity of a spit leading to the westernmost island which has now largely vanished. There is also evidence of a recent breakwater, built in 2016 on top of a pre-existing protrusion of modern rubble (Fig 9B). To the northeast, rates of change have been relatively low, and suggest mainly stability/advance with only one major area of change located ~300 m east of the jetty (Table 5; Fig 8). VHR data from the last decade suggest increased erosion rates. However, given the uncertainties associated with use of the waterline proxy, some of this could be a product of waves and tides rather than long-term erosion (Fig 8B). This is further substantiated, firstly, by the fact that the seaward-most waterline position is not always the earliest. Secondly, the backshore scarp also exhibits relatively few transects with statistically significant erosion, which in turn suggests that it has still sufficient width of beach protecting it. The only major area of visible change is located 300 m east of the modern jetty (Fig 9B). Here the coastal scarp fronting a group of modern buildings appears to be eroding away. It cannot be ruled out that slow erosion is nibbling away at the backshore, particularly during storms, but, overall, clear retreat of the backshore is much less apparent compared to southwest of the Ptolemais headland.

#### Tocra

**Fig 10:**
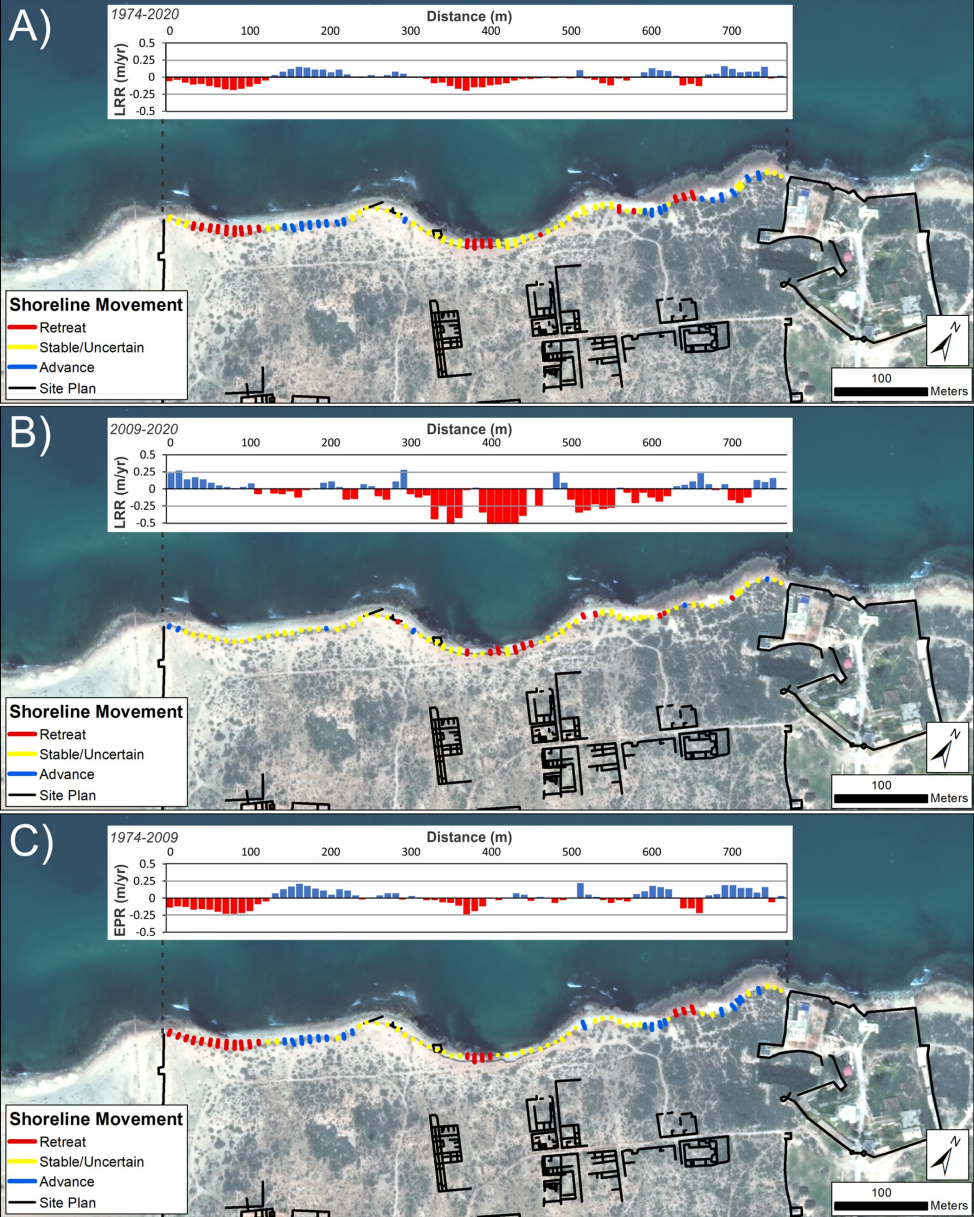
Shoreline change for Tocra (VHR images). DSAS shoreline change transects classified into statistically significant categories based on LRR and 90% LCI, or EPR and 90% ECI as obtained from VHR imagery. Quantified LRR or EPR (metres/year) is plotted on the inset graph with negative values (red) indicating erosion/retreat and positive values (blue) indicating accretion/advance. Shoreline proxy is the backshore cliff. A) Full times series: 1974–2020; B) Recent: 2010–2020 and C) Historic to recent: 1974–2010. Basemap: ©Maxar (21/03/2020), provided by European Space Imaging.

Analysis focussed on the cliffline at the ancient site where erosion and damage to archaeological structures has been reported and continues to be observed [8]. Calculated rates of change are summarized in Table 6 and mapped in Fig 10. These confirm the trend of erosion identified on the ground at Tocra but also highlight that it is not consistent across the ancient site. This supports the pattern identified from the composite Landsat shorelines (Fig 4). In fact, all the temporal categories show a roughly even split between eroding and accreting transects with a slight tendency towards erosion (Table 6; Fig 10). In almost half of the transects, the rates of change are sufficiently low that it is unclear if they show genuine change (i.e. classified as uncertain). Over the 1974–2020 interval, definitive erosion has concentrated in the western and central parts of the site, with some localized patches towards the east (Fig 10A).

**Table 6:** Summary DSAS results based on VHR imagery for Tocra.

	1974 to 2020	2009 to 2020	1974 to 2020
**End Point Rate (EPR)**			
**Total number transects**	78	78	78
**Average EPR (m/yr)**	-0.02	-0.07	0
**Average EPR confidence interval**	0.06	0.21	0.08
**EPR Erosional transects: %**	56.4	55.1	48.7
**EPR Statistically significant erosional transects: %**	33.3	18	24.4
**EPR Max erosion rate (m/yr)**	-0.18	-0.64	-0.24
**EPR Average erosion rate (m/yr)**	-0.09	-0.2	-0.1
**EPR Accretional transects: %**	43.6	44.9	51.3
**EPR Statistically significant accretional transects: %**	24.4	5.1	24.4
**EPR Max accretion rate (m/yr)**	0.16	0.29	0.22
**EPR Average accretion rate (m/yr)**	0.07	0.1	0.09
**Linear Regression Rate (LRR)**			
**Average LRR rate (m/yr)**	-0.02	-0.07	-
**Average LCI (m/yr)**	0.07	0.25	-
**LRR Erosional transects: %**	59	55.1	-
**LRR Statistically significant erosional transects: %**	28.2	15.4	-
**LRR Max erosion rate (m/yr)**	-0.2	-0.58	-
**LRR Average erosion rate (m/yr)**	-0.08	-0.21	-
**LRR Accretional transects: %**	41	44.9	-
**LRR Statistically significant accretional transects: %**	26.9	7.7	-
**LRR Max accretion rate (m/yr)**	0.16	0.28	-
**LRR Average accretion rate (m/yr)**	0.08	0.1	-

ECI: Confidence interval of EPR (90%), LCI, Confidence interval of linear regression (90%); erosional transects are those with negative LRR/EPR; accretional transects are those with positive LRR/EPR; statistically significant erosional/accretional transects are those which still have negative/positive LRR /EPR after inclusion of LCI/ECI.

Separating the time series into recent (last decade) and 1974–2019 portions also suggests additional patterns. The overall balance between erosion and accretion does not change dramatically, but the last decade appears to show a tendency towards more rapid rates of erosion, particularly concentrated in the central part of the site (Fig 10B). By contrast, 1974–2009 appears to show more erosion on the western part of the site which is no longer apparent or, at least, is much reduced (Fig 10C). The caveat is that for the last decade, larger uncertainty values reduce the number of transects definitely showing erosion.

Even so, these highlight that the historic and recent tendency has been for erosion to concentrate in the central portion of the site. Here, an open bay is gradually being cut into the exposed cliffline. There are hints of retreat on either side of this bay, but in general these areas have shown relatively little change over the past decade (Fig 10). In summary, there is clearly a trend of erosion at Tocra which has been proceeding since the 1960s, and appears to be increasing in recent years, both on the basis of calculated rates of change (Table 6; Fig 10) and eyewitness observations [8]. Further, the retreat rates of ~-0.2 m/yr estimated by [8] match those calculated here from satellite imagery.

### Future shoreline forecasts

#### Apollonia

**Fig 11:**
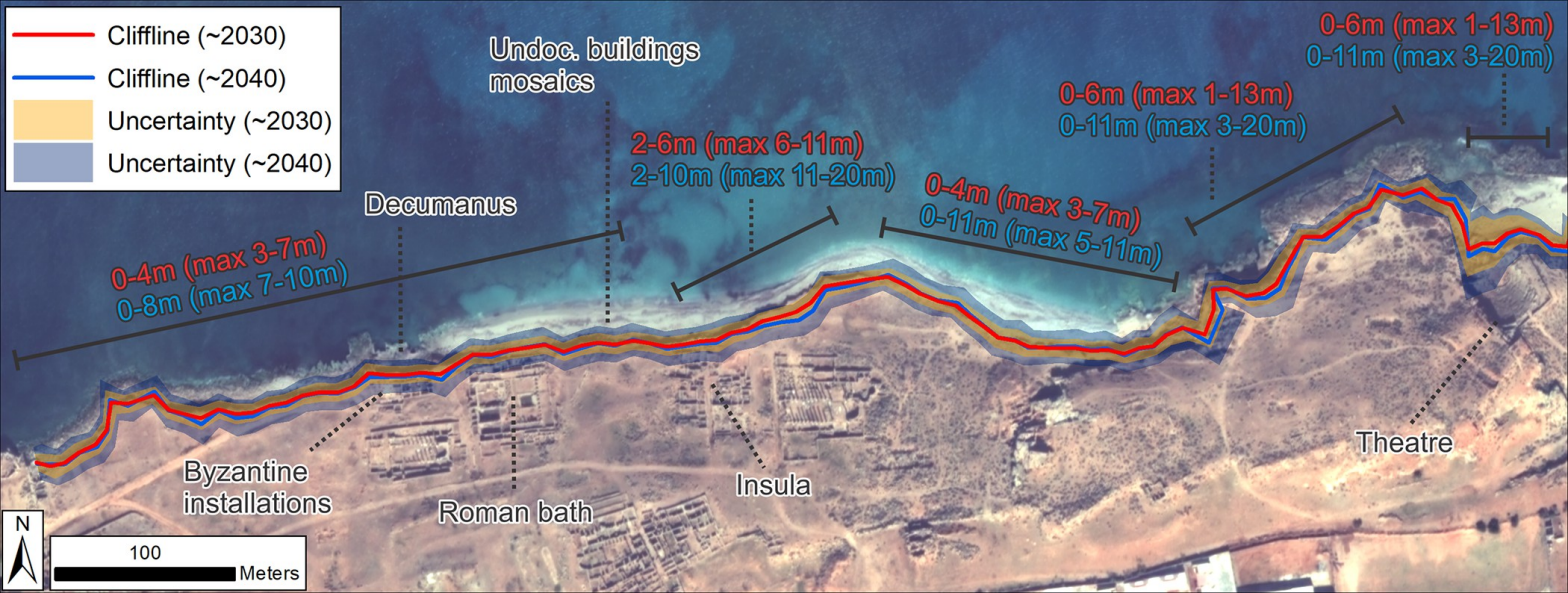
Shoreline forecasts for Apollonia based on recent LRR from VHR images. Text shows generalized magnitude of retreat for the backshore cliffline relative to 2019 for different sections of coastline and for 2032 (red) and 2042 (blue). Values only refer to erosion/retreat not advance/accretion: Zones with 0 values show where stability/advance has also been forecast. Values in brackets show the maximum retreat based on the uncertainty bands. Archaeological structures/material forecast to be at risk are annotated. Basemap: ©Maxar (7/11/2019), provided by European Space Imaging.

Forecasts for the western part of Apollonia place the 2032 and 2042 clifflines at or close to their 2019 equivalent with localised instances of projected retreat of up to 4–8 m respectively (Fig 11). These occur where indivudual transects recorded higher rates of change within the overall pattern of stability/slow retreat. If the full uncertainty values are included, they suggest that this area could, in extreme circumstances, witness retreat of the coastal edge of 7 m by 2032 or 10 m by 2042.

**Fig 12:**
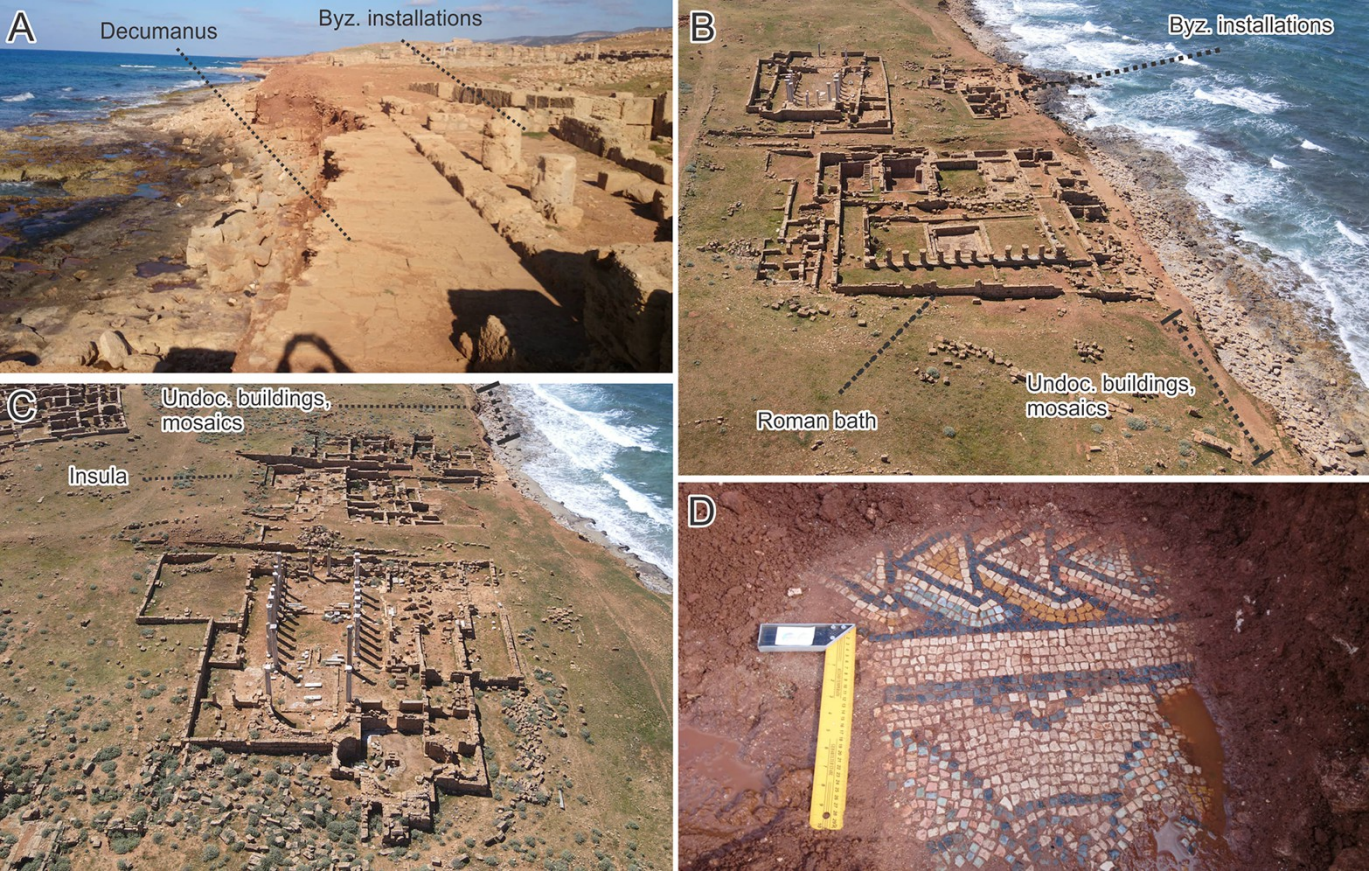
Present-day erosion impacts at Apollonia. A) Erosion of the Roman road (*decumanus*) in front of the Byzantine installations. B) eroding edge in front of the Byzantine Installations and Roman bath. C) eroding edge in front of the insula. Also indicated is the stretch of backshore where undocumented buildings and mosaics are eroding out. D) Example of a mosaic floor exposed by backshore erosion (photos A and D: 2019, S. Buyadem; photos B and C: 2021, F. El-Gumati).

Nevertheless, the proximity of archaeological structures at, or within a few metres from the eroding backshore means that even relatively low rates of retreat (e.g. <-0.2 m/yr) present a major threat. One of Apollonia’s major thoroughfares, probably the *decumanus*, runs parallel to the existing coastline. This road has already been severely damaged by wave action (Fig 12A). The sections cut by ongoing erosion have revealed a series of earlier road surfaces underneath the paved Roman road. Further erosion would see large parts of this road fully worn away, particularly in front of the Byzantine installations and the Roman bath (Fig 12B). The Byzantine installations themselves would likely also suffer considerable damage especially if the upper end of the uncertainty values are considered. The function of the Byzantine installations has yet to be fully determined, as they have not yet been entirely excavated. It is likely that there was, first, a Roman bathhouse that was converted into a residence or processing facilities during the Byzantine period. Immediately to the east of the Byzantine installations are the remains of the Roman bath. Deep excavations revealed votive deposits from as early as the fourth century BC and an earlier peristyle structure. The Roman bath had a long lifespan; it was in use to some extent until the sixth and seventh century AD and has undergone several alterations. The areas of the bath most likely affected by coastal erosion over the next 20 years include the entrance to the bath, the frigidarium, the latrines, the aqueduct and part of the peristyle [3].

The western side of the incipient tombolo is forecast to retreat by 2–6 m by 2032 and 2–10 m by 2042. Again, if the full uncertainty range is used, these respectively increase up to 11 m and 20 m. Conversely, the eastern side of the tombolo is forecast to remain stable or even advance by up to 2 m (2032) to 4 m (2042). Nevertheless, inclusion of uncertainty values suggest retreat of up to 4 m (2032) to 8 m (2042) is still possible. Immediately west of the tombolo lies an *insula* with a series of buildings arranged left and right of a road running north-south, away from the *decumanus* (Fig 12C). Little is known about this area, because the results of an excavation by a French expedition in 1954–55 were unfortunately never published. Some vats suggest industrial activity in this otherwise mainly residential area. Based on the shoreline forecast, the north-western edge of this *insula* will suffer from erosion over the next 20 years. An area of approximately 500 m wide between the Roman bath and the *insula* remains unexcavated, but traces of buildings can be seen in the eroding face of the backshore, including several layers of mosaic floors (Fig 12C and 12D). These too will be lost if the shoreline retreats as forecast.

The archaeological implications for the main body of the tombolo are more unclear as no excavations have been undertaken in this area. However, from the section of the backshore exposed by erosion it is clear that buildings with substantial walls once stood in this location. The eroding edge on the steeper slope to the east of the tombolo is forecast to locally retreat by up to 6 m (2032) to 11 m (2042). Inclusion of uncertainty values increases this to up 13 m (2032) and 20 m (2042). However, whether these values could be achieved in reality is uncertain. The steep slopes and bedrock, which lies close to the surface in this area, would more likely keep retreat rates to the lower end of the forecast.

Finally, at the eastern end of the site, forecast retreat is 1–8 m by 2032 or 2–11 m by 2042. Wide uncertainty bands increase this to 22 m for 2032 and 34 m by 2042. Whilst these values appear alarming, the caveat is that this area comprises an unconsolidated beach berm rather than an eroding cliff. Its future response is therefore much more uncertain. This area contains the theatre, which was constructed in the Hellenistic period and later modified: the stage was altered and brought forward towards the end of the first century AD, while the stage building behind was replaced by a larger structure. The forecast data raise the strong possibility that the remains of the stage and stage building will probably be most affected by coastal change over the next 20 years. This might not necessarily be erosion but could also include partial burial by sand washed over from the berm. That change will happen within the next 10–20 years is supported by eyewitness accounts which confirm that the sea already reaches the back walls of the stage during winter storms.

#### Ptolemais

The vast majority of work at Ptolemais has focussed on the inland part of the site with only limited survey of the submerged harbour and a handful of test excavations on the coast [3,46,88]. Consequently, the location and significance of archaeological features on the coastal edge are poorly documented. This is now changing with the initiation of the CCS [5]. Analysis is still in progress, but preliminary results indicate the presence of vulnerable archaeological material on, or close to, the eroding backshore.

**Fig 13:**
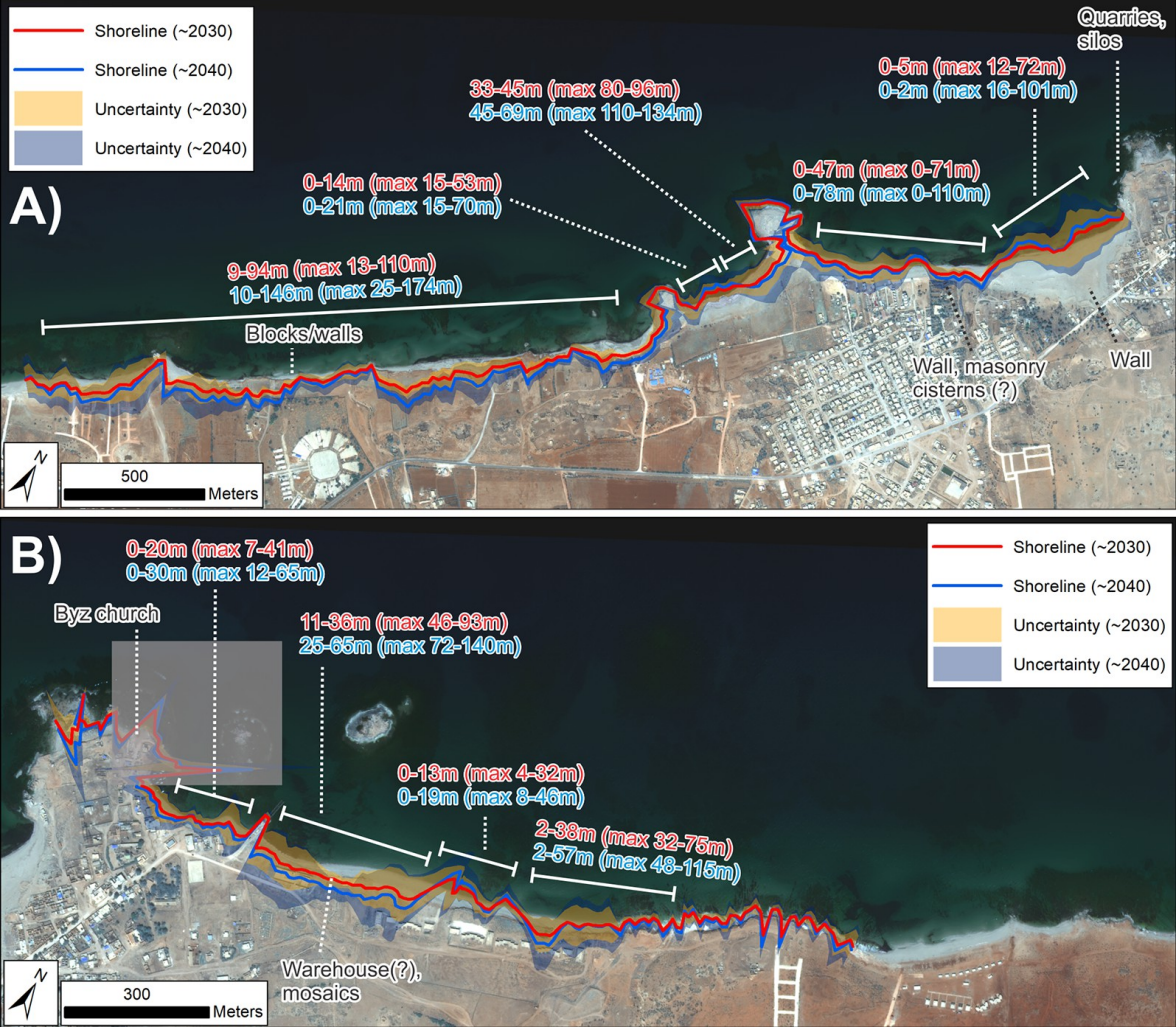
Shoreline forecasts for Ptolemais based on recent LRR from VHR images. A) area southwest and B) northeast of Ptolemais headland. Text shows generalized magnitude of retreat for the backshore cliffline relative to 2019 for different sections of coastline and for 2032 (red) and 2042 (blue). Values only refer to erosion/retreat not advance/accretion: Zones with 0 values show where stability/advance has also been forecast. Values in brackets show the maximum retreat based on the uncertainty bands. Archaeological structures/material forecast to be at risk are also annotated. Basemap: ©Maxar (25/04/2016), provided by European Space Imaging.

**Fig 14:**
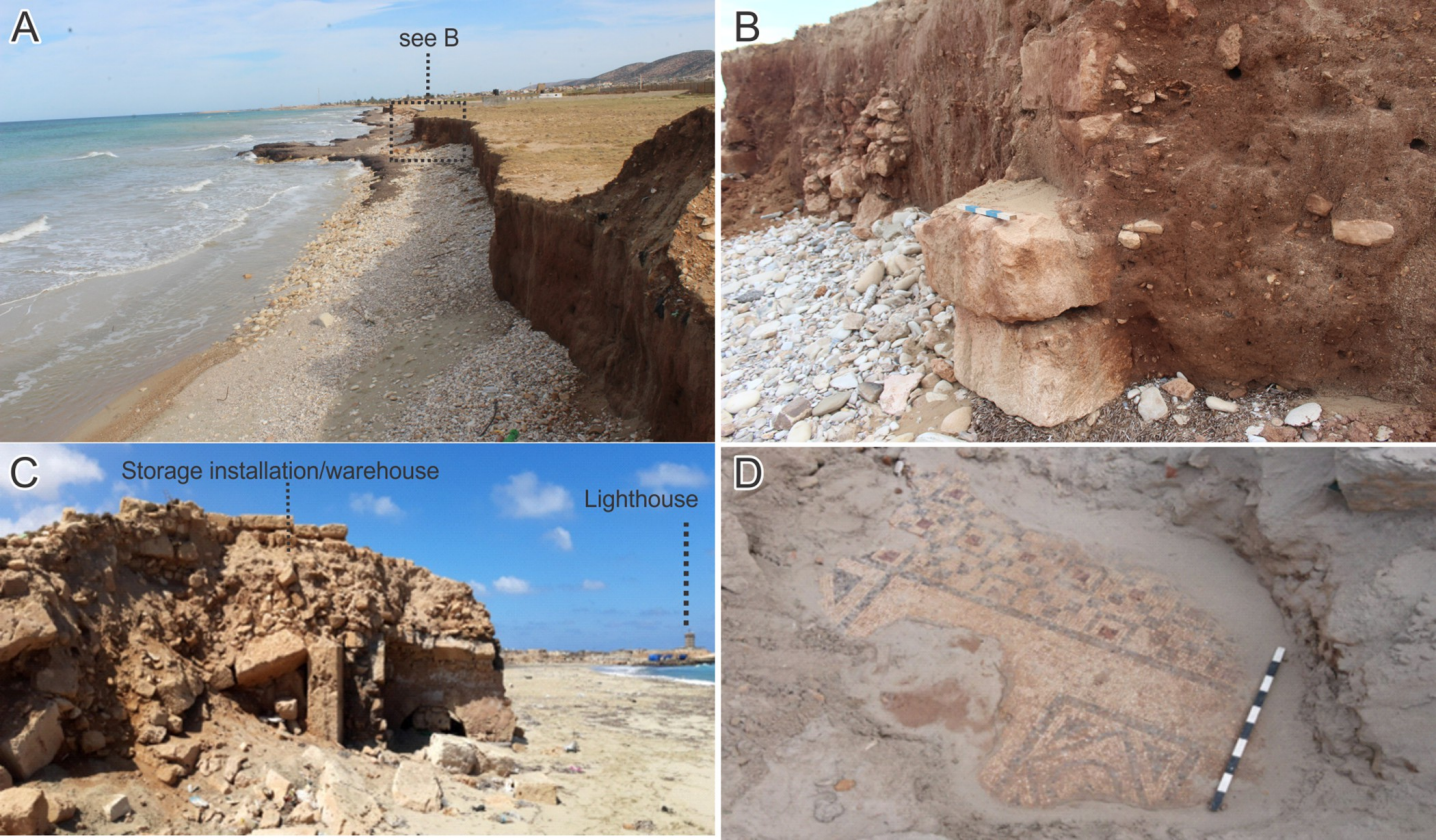
Present-day erosion impacts at and near Ptolemais. A) Eroding cliffline west of Ptolemais. B) Blocks/structural material eroding out of the cliffline. C) industrial features (warehouses?) exposed by wave action east of the Ptolemais headland. D) Exposed mosaic floors east of the headland (photos A and B 2021: CCS Survey; photos C and D 2022: S. Buyadem).

For the initial stretch between the westernmost edge of the study zone and the first small headland, the forecast 2032 shoreline lies 9–94 m inland of the 2016 waterline, and the forecast 2042 shoreline 10–146 m inland (Fig 13A). Including uncertainties increases the magnitude of possible retreat to 110 m (2032) and 174 m (2042). Observed erosion here is considerable, with a clear vertical face cut into the unconsolidated cliff. Blocks and structural remains are eroding out of the cliff and, given the forecast coastal retreat, will be destroyed in the next few years. The extent of retreat also indicates that if these features connect to more material further inland, this too will likely be exposed within the next decade (Fig 14A and 14B).

At the first small headland east of this eroding stretch, forecast retreat magnitude falls significantly and the 2032 and 2042 shorelines largely coincide with, or lie within a few metres of, the 2016 waterline. Immediately east of this headland, progradation of up to 28 m (2032) to 47 m (2042) is forecast for the first half of the beach. However, this does not rule out erosion; inclusion of uncertainties suggests that retreat of up to 53 m (2032) and 70 m (2042) could occur. The second half of the beach is characterised by forecast retreat of up to 45 m (2032) to 69 m (2042). Uncertainty-induced maximum retreat values are 96 m (2032) and 134 m (2042). At the next headland, forecast retreat is again minimal, and largely occurs only if uncertainty values are included (Fig 13A). However, the pattern of change on the adjacent beaches suggest the liklihood that the tomoblo will narrow and eventually be cut through entirely, severing the headland from the mainland.

The final beach preceding the Ptolemais headland also displays variable forecasts. Its western half is projected to retreat by up to 47 m (2032) to 78 m (2042), but its eastern half is forecast to advance by several tens of metres. Again though, the inherent uncertainty suggests that retreat is still possible for all sections of the beach. This beach contains the remains of a substantial wall (the so-called ‘Beecheys’ wall first documented in the early 1800s: [46]). The position of this wall is such that most of its eastern end lies inland of forecast retreat for the next 20 years (Fig 13A). However, its western end, and material such as ashlar masonry, walls and fragmentary cisterns located to its west, lie within an area of forecast erosion. More vulnerable material such as industrial installations and production sites (tanks, vats/silos, quarries) lie within or just above the intertidal zone of the western side of the Ptolemais headland [46,88], and newly-exposed and previously-undocumented substantial structures were recorded by the CCS in 2021.

For the eastern side of the Ptolemais headland, forecast values are influenced by recent breakwater construction and are therefore disregarded. The section of beach on the southeast side of the headland west of the jetty is forecast to retreat by up to 20 m (2032) to 30 m (2042) and with maximum uncertainty-driven estimates of up to 41 m (2032) and 65 m (2042). Immediately east of the jetty, retreat is also forecast: 11–36 m (2032) and 25–65 m (2042) with maxima of up to 93 m and 140 m respectively (Fig 13B). In this area, it is unclear if the city walls once separated the harbour from the shoreline settlement, as was the case at Tocra and Apollonia. This is because this section of the city’s defences could have already been claimed by the waves in the past, coupled with more recent re-use of its materials [3,89]. Even so, the area in the vicinity of the ancient harbour is rich in archaeological remains. This includes a Byzantine church on the Ptolemais headland, now covered by a modern lighthouse, and the remains of possible storage buildings (warehouses) to the east of the modern jetty (Fig 14C). Similar to the other harbours along the Cyrenaican coast, various industrial structures must have accumulated around Ptolemais’ harbour, many of which are likely covered by the modern town or, outside its present boundaries, by accumulated sand and soil. Moreover, as well as industrial installations, this area also contains evidence of domestic use in the form of buildings (possible villas) with mosaic floors (Fig 14D). Given the forecast erosion rates, much of this material will likely be lost within the next 20 years.

This area gives way to a short stretch of forecast progradation with limited localized retreat of up to 13 m by 2032 and 19 m by 2042 (Fig 13B). This in turn gives way to a short section which once again has forecast retreat (2–38 m by 2032 and 2–57 m by 2042). From this point on, the forecast shorelines lie close to the 2016 waterline. Future change is strongly mitigated by the rocky and steep nature of the coastline at this point.

#### Tocra

**Fig 15:**
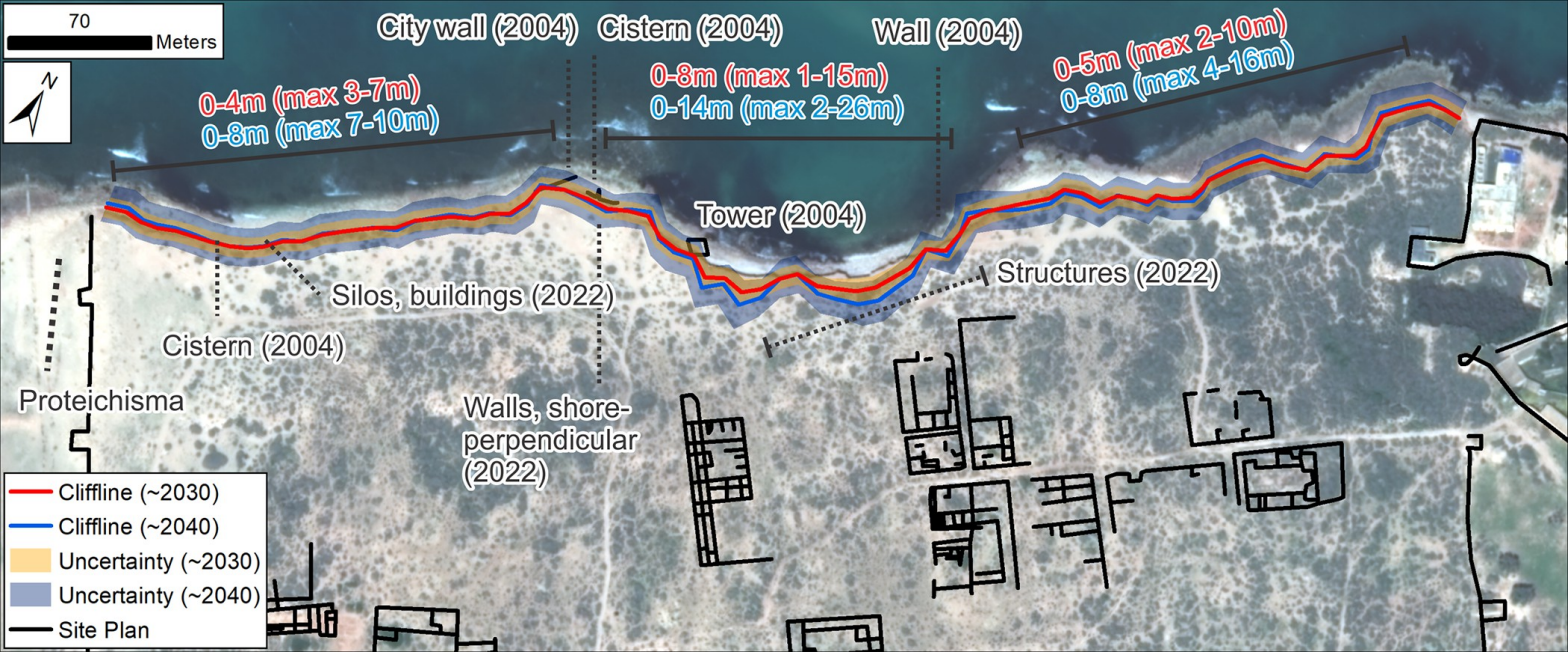
Shoreline forecasts for Tocra based on recent LRR from VHR images. Text shows generalized magnitude of retreat for the backshore cliffline relative to 2019 for different sections of coastline and for 2032 (red) and 2042 (blue). Values only refer to erosion/retreat not advance/accretion: Zones with 0 values show where stability/advance has also been forecast. Values in brackets show the maximum retreat based on the uncertainty bands. Archaeological structures/material forecast to be at risk are also annotated. Basemap: ©Maxar (21/03/2020), provided by European Space Imaging.

**Fig 16:**
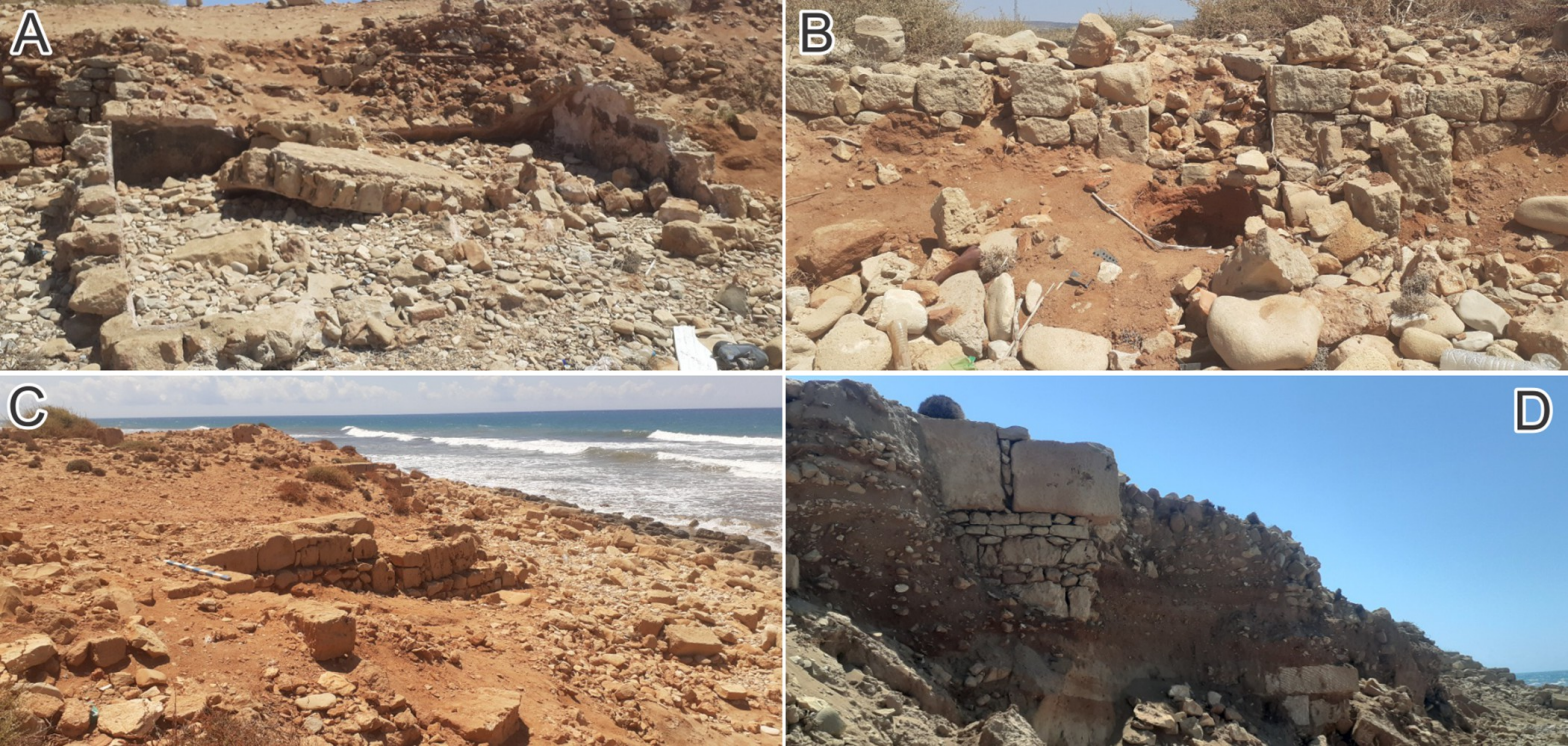
Present-day erosion impacts at Tocra. A) Roofed cistern documented in 2004; now largely destroyed. B) walls, silo and building, not visible in 2004 and since exposed by coastal erosion. C) Shore-normal walls exposed post-2004 by coastal erosion. D) Part of the defensive perimeter wall, now exposed (all photos: 2022, S. Buyadem).

The area potentially affected by future erosion is little explored and documented. From the little we do know—mainly from stratigraphy exposed by storms—this area contains defensive structures, quarries and cisterns and other industrial features. In the western part of the site, the projected 2032 and 2042 clifflines closely follow the 2020 cliffline (Fig 15). Forecast retreat is low, locally reaching up to 2 m (2032) and 3m (2042). Inclusion of uncertainties increases the projected maximum retreat to 7 m (2032) and 11 m (2042). In the westernmost part of the study area lies the *Proteichisma*, a curtain wall from the Hellenistic period which was surveyed in 2004 [8]. This wall was already damaged in 2004 and recent visits by the CCS indicate that it has suffered further erosion. A second section of wall located east of the *Proteichisma* and running parallel to the seafront was also surveyed in 2004. Even then it was reported that ‘*nothing can be seen beyond a short remnant section of tumbled stonework*’ ([8]:116). The additional recent damage to the *Proteichisma* and already-damaged nature of the shore-parallel seafront wall suggest that even with relatively low rates of forecast erosion, further damage will occur over the next 20 years. This area also included a roofed cistern, formerly subterranean but exposed by the loss of beach sediment. This has completely collapsed post-2004 and will likely be entirely eroded away by 2040 (Fig 16A). In this general area, in 2022, the CCS recorded structures—including the entrance to a building, a silo and cisterns—that were newly-exposed as a result of coastal erosion (Fig 16B). Their location right on the vulnerable eroding edge means that even with only a couple of metres of erosion over the next 20 years, their destruction is highly likely. At the junction of the east-west running cliffline and the western part of the central bay, the foundation of the shore-parallel seafront curtain wall was exposed and, already in 2004, impacted by wave action. This foundation is not apparent on recent images, and instead the CCS has documented several large shore-perpendicular walls which are eroding out of the cliff, and will mostly likely be gradually destroyed over the next 20 years (Fig 16C).

The amount of forecast erosion increases in the central part of the site: up to 8 m for 2032 and 14 m for 2042, though there are localised instances where the forecast still tracks closely the 2020 cliffline. This increases to maxima of 15 m (2032) and 26 m (2042) with inclusion of uncertainties. East of the central bay, forecast retreat reduces and the projected clifflines again closely follow the 2020 cliffline with localized retreat of up to 5 m (2032) and 8 m (2042) and uncertainty-driven maxima of 10 m (2032) and 16 m (2042). In the central area, the 2004 survey identified a second cistern, already breaking up due to wave action, and the remains of an Archaic period wall tower, also already undergoing damage. The exposed section of the cliff to the east of the tower also contained the eroding-out remains of various buildings/walls [8]. A further section of the Archaic wall was exposed 120 m east of the tower and, in 2004, had already been cut down to its basal courses. The CCS has documented within this area a number of structures and possibly part of the former defensive perimeter wall eroding out of the cliff (Fig 16D) which, given the forecast rate of erosion, will almost certainly be heavily damaged over the next 10 years. Moreover, the continual exposure of archaeological material since the 1960s suggests that the area immediately inland of the eroding cliffline contains numerous buried structures which have minimal exposure on the clifftop surface. Continued erosion at current rates will therefore almost certainly lead to the exposure and destruction of additional undocumented material in the coming years.

## Discussion

### Controls on coastal change

The shoreline change assessment has demonstrated the location and magnitude of coastal erosion within the study area. That this is evident from the application of the same approach to two independent imagery time-series at different resolutions strongly suggests that the identified spatial pattern of change is robust. In addition, use of a VHR imagery time-series incorporating recent and historic images has also highlighted the possibility that the rate of erosion has increased in recent years. It is important to understand the processes which have caused these changes. This will enable consideration of whether the forecasts presented here are valid, which management solutions are best for the future and whether similar impacts will occur elsewhere along this coastline.

In general terms, coastal change is dependent on multiple factors, not least the wind-wave climate, tidal regime, coastal orientation, resistance of coastal rocks, changes in RSL and sediment supply. On the Cyrenaican coast, tides are minimal and their impact can be largely disregarded. However, waves exert a particularly strong impact. The dominant north and northwesterly winds combined with large fetch and relatively narrow continental shelf (particularly between Ptolemais and Apollonia) ensure that significant (*Hs*) and maximum (*Hmax*) wave heights are among the highest on the North African coast, especially during autumn, winter and spring [19]. Their impact on archaeological sites here has also been exacerbated by RSL rise; specifically 2–3 m of submergence which occurred sometime after the establishment of harbours such as Ptolemais and Apollonia. This had the effect of bringing structures, which were originally built above water, down into the destructive surf zone. In short, these two factors, namely a strong wave climate coupled with extensive archaeological remains within the zone of wave attack ensures a particularly high risk for the archaeology along this coastline.

However, if these were the only factors influencing the preservation of coastal archaeological sites in Cyrenaica then we would expect far less survival. For example, using a relatively conservative calculation, such as erosion rates of -0.1 to -0.5m/yr (i.e. the lower end of the LRR values for the study sites) over a period of 1000 years, the expected coastal retreat would be 100–500 m. Given that Ptolemais, Apollonia and, to a lesser extent Tocra, were all working harbours, with buildings down to the shore, if such rates had prevailed since their establishment, a much larger proportion of each site would now be eroded away. This assertion is also substantiated by the comparison between the Beechey survey [6] and modern imagery which indicates that large parts of the Apollonia shore have not changed dramatically in the last 200 years, and eyewitness accounts which indicate that some archaeological remains, such as the Western Gate tower, have not changed from the original 1958 survey to the present day.

Consequently, site survival here has also been influenced by other factors. At Apollonia and Ptolemais, the nearshore reefs and ridges of aeolianite (which were also instrumental in the selection of these locations as sheltered harbours) have undoubtedly served as natural breakwaters. This effect is less certain for Tocra, since the only ridges here are fully submerged. However, some protection was presumably once offered by artificial breakwaters, such as were recorded by dive surveys in the 1960s [90]. In addition, the position of Tocra, and to a lesser extent, Ptolemais, on the southwestwards curving shore of Cyrenaica, raises the possibility that they also benefited from longshore accumulation of sediment generated by the dominant northerly winds.

The question, therefore, is what has now changed, to reduce these protective effects? One factor could be natural weathering of the aeolianite ridges. At Apollonia for example, a section of wall on the West Island, which had been created by quarrying, was destroyed by the early 1960s [4]. The cause of the collapse is not definite, but was probably wave action. Similar effects have not yet been documented at Ptolemais, while at Tocra, the fact that the artificial quays/breakwater are fully submerged would imply either their submergence by RSL rise, or their natural breakdown over the centuries post-abandonment of the harbour. Nevertheless, while such breakdown could have long-term consequences for site survival, whether it would lead to similar patterns of accelerated erosion at all three sites in the same time period is far less certain. It is more likely to have variable and localized implications. For example, at Apollonia, although the wall has collapsed, the underlying reef is still present and thus the overall pattern of wave breaking and refraction probably not changed on this account.

**Fig 17:**
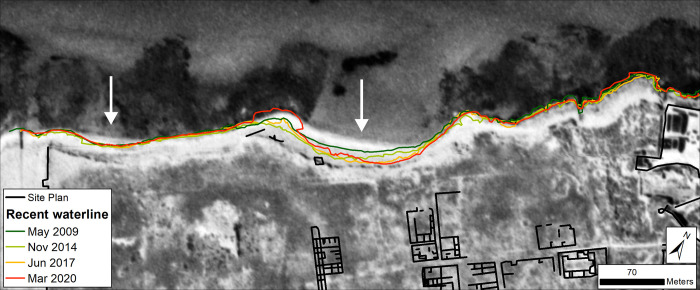
Comparison of Tocra in 1974 from KH-9 image with waterlines from the last decade superimposed. Note the near total loss of the beach in the central part of the site and reduction of the beach in the western part (both indicated by white arrows). Outside these two areas, the remaining waterlines are broadly stable with slight differences resulting from waves/tides at the time of image acquisition. KH-9 image provided by USGS [80].

A more probable factor is a reduction in sediment supply. For example, at Tocra, comparison of the 1974 and recent VHR images reveal that the central and western portions of the site (i.e. those with the highest erosion rates) were once fronted by beaches (Fig 17). The central beach appears to have been present up to 2009 since when it has been almost entirely removed. This enables waves to reach right up to the unconsolidated backshore thereby accelerating its erosion. Similarly, at Ptolemais, the areas with the most extensive erosion are those which were once fronted by an extensive beach (Fig 9A). At the ancient site of Apollonia, extensive changes in beach width are not evident over the VHR image time series, but similar beach loss appears to have affected an area of extensive erosion located c. 2–3km west of the ancient site (Fig 2).

Evidence from elsewhere across North Africa suggests that 20^th^ century beach loss is not an isolated occurrence and that the key drivers are anthropogenic; principally a reduction of sediment supply via upstream damming and urbanization [17,18]. Comparison of the 1974 KH-9 with recent VHR images demonstrates increased urbanization across the coastal plain of Cyrenaica and construction or enhancement of roads which directly cross many of the seasonal wadis (see also [5,59]). The resultant culverting, damming or diversion of these watercourses would likely lead to some reduction in sediment delivery.

However, an additional and, in certain areas, more significant driver of reduced sediment supply is probably coastal sand mining. This involves removal of beach sand, generally for the construction industry. Rapid urbanization over the latter half of the 20^th^ century has led to an increasing demand for sand, and hence an increase in sand mining. This activity is often unregulated, illegal and often goes undocumented. However, its environmental impacts are far-reaching, and include beach loss which results in reduced protection from waves and storms, and hence accelerated coastal erosion [91–93]. These effects have been reported elsewhere in North Africa—for instance Morocco and Algeria [94,95] —but Libya is presently absent from a global database of coastal sand mining [96]. This, however, is probably due to a lack of reporting rather than the actual absence of mining. Within the study area, sand mining is evidenced by buildings and vehicle tracks visible on the 1974 and recent VHR images on the beach the southwest of Ptolemais (Fig 9). The 1974 evidence suggest that mining may have been ongoing intermittently in some places for several decades, but the recent (late 20^th^ to early 21^st^ century) increase in erosion rates suggests it may have increased in recent years, probably linked to increasing urbanization and development [5,97]. Field observations of unauthorized sand mining have also been made at various locations southwest of Ptolemais and elsewhere on the coastline between Tocra and Apollonia [5,98].

### Implications for future preservation and management

If the above are the primary causes for accelerated coastal erosion in Cyrenaica, we see no reason why they will reverse in the immediate future and significantly alter the forecast patterns. Weathering and breakdown of protective ridges will only continue with time, and recent fieldwork highlights the increasing trend of urbanization, development and building (often unregulated) in this region [5]. Therefore, there is no reason to suspect that sediment delivery to the coast will increase. Some localized reduction of erosion could occur if coastal sand mining was halted, particularly given the recognition of its destructive impacts on other aspects of the environment, such as coastal water quality [98]. Achieving this would probably require regulation or legislation, which in turn is presumably dependent on local socio-economic and political pressure. Consequently, whether this would actually happen is hard to predict. Nevertheless, efforts should be made, perhaps using approaches such as presented here, to raise awareness in local communities and put pressure on local authorities to recognize the destructive impact of sand mining, not only on archaeological sites, but also the wider environment and, eventually, homes and infrastructure.

Assuming that present-day rates of erosion will continue, extensive stretches of coastline at the important archaeological sites of Tocra, Apollonia and Ptolemais are at risk. This is not to say that each site will be lost in its entirety but the coming decades will see progressive loss and damage to individual structures and their associated material culture. The situation could also worsen with climate change [99]. Future projections show the likelihood that sea-level will rise over the 21^st^ century with an acceleration post-2050 if greenhouse gas emissions remain high [12,15,100]. This will allow waves to pass more easily over the outlying reefs and thus reach the shore with greater force. Increased RSL elevation combined with the absence/reduction of beaches will also allow waves to strike more frequently at the vulnerable unconsolidated backshore. Moreover, although Mediterranean-wide climate projections forecast a reduction in storm intensity for the wider region, it is worth noting that hindcast models have shown that Cyrenaica has actually experienced a slight increase in summer significant wave weight during the 20^th^ century [19]. If this continues, and when combined with the already strong local wave regime, the implication is that larger, more destructive, waves could occur in all seasons.

One of the primary concerns is that archaeological sites and features located directly at the shoreline, and outside ancient walled perimeters, are understudied and undocumented. So far, the primary focus has either been on the already submerged harbour features (e.g. Apollonia, Ptolemais), or on elements of the ancient town that are located further inland and inside the city walls. This study demonstrates the urgency for thorough documentation to preserve invaluable information about Libya’s past, especially with regard to its involvement in Mediterranean trade networks during the Classical periods and beyond. That said, it is not only Classical features that are at risk. The harbours and settlements along the coast often continued to function into the Arab periods, although at a smaller scale. The more recent Ottoman and Italian occupations of Libya in the 19^th^ to the mid-20^th^ century left behind forts, buildings and other harbour installations that are also being claimed by the sea, such as the Italian fortress at Tocra, which has been severely damaged by the waves over recent years.

The importance of developing detailed management and mitigation plans for coastal sites cannot be stressed enough. We have to accept that physical protection for archaeological sites (e.g. seawalls, rock armour) may be cost-prohibitive given the number of vulnerable sites on the Cyrenaican shoreline, and also would have to be future-proofed against future SLR. Moreover, hard engineering solutions may also be inadvisable considering the sensitive nature of the local sedimentary regime, as demonstrated by the effect of sand mining. Therefore, the likelihood of ‘active preservation’ will be on a case-by-case basis and probably only for the most significant or valuable sites (which itself must be determined in consultation with local stakeholders) [101]. Most likely, it will have to be accepted that some sites or parts of these sites will be lost (see for example [102]). However, this does not mean a ’do nothing’ approach. Rather, mitigation should include detailed investigations, survey and documentation of the areas that will be lost to the sea forever over the next decades.

At the time of writing, the capacity for maritime archaeology in Libya is in need of further development. Efforts to establish a maritime unit at the Department of Antiquities were undertaken before the Arab Spring in 2011, but were hampered by the civil unrest which followed. A maritime unit was finally established in 2017, but capacity is still relatively low. Over recent years, many foreign missions have successfully focused on capacity building in Libya [103–107], but maritime skills have yet to be developed further. Due to the urgency of the situation along the coast caused by coastal erosion, such capacity building, together with documentation projects, must be at the top of the agenda.

## Conclusion

Coastal erosion is not a new problem on the coast of Cyrenaica. However detailed analysis of shoreline changes extracted from satellite imagery suggests, firstly, that it is widespread and, secondly, that it has accelerated in recent years. The likelihood is that this has been caused by human actions, principally (illegal) coastal sand mining and urbanization. Importantly, the region contains many archaeological sites, many of which are understudied or not fully documented. The vulnerability of these sites to erosion has been demonstrated here by case studies of the ancient harbours of Apollonia, Ptolemais and Tocra. Erosion impacts at these sites are evidenced by both satellite images which show clear evidence of shoreline retreat, and field observations from the CCS team which have identified newly-exposed structures and destruction of, or damage to, previously-documented ones. Forecasts of shoreline change—based on present day rates of shoreline movement derived from the satellite imagery analysis—further emphasize the progressive loss and destruction of invaluable archaeological material over the next two decades. Worryingly, we stress that what is going on at these key sites is not an isolated occurrence. Both the evidence from Landsat imagery and from field observations show that this reflects the situation along the Cyrenaica coast at large. Many more undocumented or less well-studied sites are also presently undergoing erosion or will be at risk from erosion in future. This latter risk could also potentially increase with climate change-driven SLR, particularly if destructive activities such as coastal sand mining are not halted. Moreover, Cyrenaica is far from alone in this regard. Accelerated erosion is regarded as a global problem for coastal archaeology, related to both human action (e.g. sand mining, port development) and climate change [99] and there are examples of its destructive capacity elsewhere in the Eastern Mediterranean, such as southwest Cyprus [70], Israel [108,109] and Gaza [110]. The urgency of the situation, with sites at risk now, rather than projected to be at risk by the mid- or end-century, requires development of detailed management and mitigation plans sooner rather than later. Various actions can be proposed as part of these including rapid documentation to record material where loss is already occurring or is likely to occur in future; raising awareness among local communities about the every-day effects of climate change and environmentally problematic actions such as sand mining; and further research to characterise the rate and magnitude of erosion elsewhere on the coast, especially in the vicinity of less well-documented sites.

## Supporting information

S1 File Supporting information on shoreline extraction. This document provides additional information on the Landsat- shoreline extraction method using Google Earth Engine, including a link to the script used.
(PDF)

S2 File Supplementary results. This file contains DSAS inputs and results generated from the analysis presented here for each of the study sites.
(ZIP)
