# Supplementary material for: The impact of coastal erosion on the archaeology of the Cyrenaican coast of Eastern Libya
Source: PLoS One. 2023 Apr 12;18(4):e0283703. doi: 10.1371/journal.pone.0283703 (PMC10096274; doi:10.1371/journal.pone.0283703)
Supplement: S1 File — This document provides additional information on the Landsat- shoreline extraction method using Google Earth Engine, including a link to the script used. (PDF) [file pone.0283703.s001.pdf]

The impact of coastal erosion on the archaeology of the Cyrenaican coast of Eastern Libya

Kieran Westley[1*], Julia Nikolaus[1], Ahmad Emrage[2], Nic Flemming[3] and Andrew Cooper[1]

[1]School of Geography & Environmental Sciences, Ulster University, Coleraine, United Kingdom
[2]Dept. of Archaeology, University of Benghazi, Benghazi, Libya
[3]Independent Researcher
*Corresponding author
Email: kl.westley@ulster.ac.uk

S1 Supporting Information – Landsat Shoreline Extraction

This section provides additional detail on the shoreline extraction method applied to Landsat data as used in this study of shoreline change on the coast of Cyrenaica (Eastern Libya). The basic principle underpinning the approach is well-established. It involves:

1. Transformation of a multispectral satellite image into a spectral index geared towards water detection.
2. Thresholding of the spectral index image into a binary thematic classification showing water and non-water pixels.
3. Vectorization of the classified image in order to isolate the boundary between water and non-water pixels (i.e. the shoreline when applied to coastal areas).
4. Export of the vectorized shoreline/land-water boundary for further analysis (e.g. using the Digital Shoreline Analysis system [DSAS]).

There are multiple examples of this type of approach, or variants thereof in the literature (e.g. [1–6]). Key differences between applications of this type approach include choice of imagery, spectral index and thresholding method. For this study we used the following:

- Landsat 5 TM, Landsat 7 ETM+ and Landsat 8 OLI Collection 1 Tier 1 Level 2 (Surface Reflectance) imagery, filtered and transformed into annual composite images using a 15th percentile reducer. Filtering parameters used were:
    - Acquisition dates from 1985 to 2020 inclusive.
    - Only images which intersected polygons covering the study sites.
    - Cloud cover <25%.
    - Geometric RMSE < 10 m.
- Modified Normalized Difference Water Index (mNDWI) [7].
- Dynamic Thresholding [4].
- Export of vectorized shorelines to GIS.

All above steps in the processing chain were implemented in a Google Earth Engine (GEE: [8]) script which can be found here:

https://code.earthengine.google.com/c26e6e5dd9df008b554af95ce9b31f9e

Note: the script shown here is the version which was used in this study. This analysis was done in 2021 and therefore used the Landsat Collection 1 dataset which was current at the time but has since been superseded by the Landsat Collection 2 dataset.

In a final step, exported vector shorelines were imported to GIS software (QGIS 3.20) where they were manually checked for false detections (e.g. caused by cloud shadow) and cleaned. A Gaussian smoothing function was then employed to reduce their pixelated appearance. Rate of change statistics for the finalized shorelines were then computed using the DSAS 5.1 add-in for ArcGIS [9].

References

1. Vos K, Splinter KD, Harley MD, Simmons JA, Turner IL. CoastSat: A Google Earth Engine-enabled Python toolkit to extract shorelines from publicly available satellite imagery. Environmental Modelling and Software. 2019;122: 104528. doi:10.1016/j.envsoft.2019.104528

2. Luijendijk A, Hagenaars G, Ranasinghe R, Baart F, Donchyts G, Aarninkhof S. The State of the World's Beaches. Sci Rep. 2018;8: 6641. doi:10.1038/s41598-018-24630-6

3. Konlechner TM, Kennedy DM, O'Grady JJ, Leach C, Ranasinghe R, Carvalho RC, et al. Mapping spatial variability in shoreline change hotspots from satellite data; a case study in southeast Australia. Estuar Coast Shelf Sci. 2020;246: 107018. doi:10.1016/j.ecss.2020.107018

4. Donchyts G, Schellekens J, Winsemius HC, Eisemann E, Van de Giesen N. A 30 m Resolution Surface Water Mask Including Estimation of Positional and Thematic Differences Using Landsat 8, SRTM and OpenStreetMap: A Case Study in the Murray-Darling Basin, Australia. Remote Sens (Basel). 2016;8: 386. doi:doi.org/10.3390/rs8050386

5. Guneroglu A. Coastal changes and land use alteration on Northeastern part of Turkey. Ocean Coast Manag. 2015;118: 225–233. doi:10.1016/j.ocecoaman.2015.06.019

6. Jiang W, Ni Y, Pang Z, Li X, Ju H, He G, et al. An Effective Water Body Extraction Method with New Water Index for Sentinel-2 Imagery. Water 2021, Vol 13, Page 1647. 2021;13: 1647. doi:10.3390/W13121647

7. Xu H. Modification of normalised difference water index (NDWI) to enhance open water features in remotely sensed imagery. Int J Remote Sens. 2006;27: 3025–3033. doi:10.1080/01431160600589179

8. Gorelick N, Hancher M, Dixon M, Ilyushchenko S, Thau D, Moore R. Google Earth Engine: Planetary-scale geospatial analysis for everyone. Remote Sens Environ. 2017;202: 18–27. doi:10.1016/j.rse.2017.06.031

9. Himmelstoss E, Henderson R, Kratzmann M, Farris A. Digital Shoreline Analysis System (DSAS) Version 5.0 User Guide. Open-File Report 2018-1179. 2018;1179.
