# Supplementary material for: The impact of coastal erosion on the archaeology of the Cyrenaican coast of Eastern Libya
Source: PLoS One. 2023 Apr 12;18(4):e0283703. doi: 10.1371/journal.pone.0283703 (PMC10096274; doi:10.1371/journal.pone.0283703)
Supplement: S2 File — This file contains DSAS inputs and results generated from the analysis presented here for each of the study sites. (ZIP) [file pone.0283703.s002.zip › S2_SupplementaryResults.pdf]

The impact of coastal erosion on the archaeology of the Cyrenaican coast of Eastern Libya

Kieran Westley[1*], Julia Nikolaus[1], Ahmad Emrage[2], Nic Flemming[3] and Andrew Cooper[1]

[1]School of Geography & Environmental Sciences, Ulster University, Coleraine, United Kingdom
[2]Dept. of Archaeology, University of Benghazi, Benghazi, Libya
[3]Independent Researcher
*Corresponding author
Email: kl.westley@ulster.ac.uk

S2 Supplementary Results

This section provides information on the digital supplementary results files which are included with this paper. These can be divided into two groups:

1) DSAS Inputs:

This comprises digitized shorelines, transects and baselines which are required for DSAS analysis. All files are contained within personal geodatabases (.mdb); the format required for input to DSAS. One geodatabase is provided per site. Note the following file name conventions and abbreviations:

- Site names:
  - Apol = Apollonia.
  - Ptol = Ptolemais.
  - Toc = Tocra.
- Baselines: all files prefixed “baseline”.
  - Files with “Area” = used for Landsat analysis of wider area.
  - Files with “Site” = used for VHR analysis at site scale.
- Digitized shorelines:
  - Files with “DynThres” = extracted shorelines from Landsat annual composites.
  - Files with “VHR” = shorelines manually digitized from VHR imagery. Note that different shoreline types may exist within each file for the same date – e.g. a waterline as well as the backshore cliff.
- Transects: all files prefixed “Tr”.
  - Files with “Area” = used for Landsat analysis of wider area.
  - Files with “Site” or “St” = used for VHR analysis at site scale.
    - For Ptolemais, VHR analysis was broken down into three areas (A1, A2: west of Ptolemais headland; A3: east of Ptolomeis headland) respectively.
  - Files with “Cl” = used for analysis of backshore cliff.
  - Files with “W” = used for analysis of waterline.
  - Numbering convention shows values used in DSAS transect casting dialogue: baseline buffer-transect spacing-transect smoothing.
    - E.g.: Tr_Area_50b50s50s = Transects for the Landsat area analysis cast using a 50m baseline buffer, 50m transect spacing and 50m smoothing.

2) DSAS Results:

This comprises the output of DSAS analysis, namely transects with calculated rate of change statistics and forecast future shorelines and associated uncertainty bands. For ease of transfer, one file geodatabase (.gdb) is provided per site. Note the following file name conventions and abbreviations:

- Site names:
  - Apol = Apollonia.
  - Ptol = Ptolemais.
  - Toc = Tocra.
- Shorelines used:
  - Files with "DynThres" = results from extracted shorelines from Landsat annual composites.
  - Files with "OR2A" = results from shorelines digitised from recent VHR imagery only.
  - Files with "HistToRecent" = results from earliest digitized shoreline to earliest recent VHR shoreline.
  - Files with combinations of "OR2A" plus "Hunt" and/or "KH9" = results from all digitized VHR shorelines.
- Intersects: all files with "intersect".
- Rates: all files with "rates".
  - Files with suffix "clip" have been clipped in DSAS using the Shoreline Change Envelope (SCE). These files have additional fields added which apply the calculated uncertainties to the output rates and enable classification of shorelines into Accretion/Advance, Erosion/Retreat or Stable/Uncertain categories (ShoreChange field).
- Area covered:
  - Files with "Area" = results from Landsat analysis of wider area.
  - Files with "Site" = results from VHR analysis at site scale.
    - For Ptolemais, VHR analysis was broken down into three areas (A1, A2: west of Ptolemais headland; A3: east of Ptolomeis headland) respectively.
  - Files with "Cliff" = results from analysis of backshore cliff.
  - Files with "W" = results from analysis of waterline.
- Forecasts: all files with "forecast".
  - 10 year forecast: suffix "10".
  - 20 year forecast: suffix "20".
  - Forecast uncertainty bands: files with "forecast_uncy".
- Numbering convention shows values used in DSAS transect casting dialogue: baseline buffer-transect spacing-transect smoothing.
  - E.g.: 50b50s50s = Transects cast using a 50m baseline buffer, 50m transect spacing and 50m smoothing.
